# Nanotechnology Driven Innovations in Modern Pharmaceutics: Therapeutics, Imaging, and Regeneration

**DOI:** 10.3390/nano15221733

**Published:** 2025-11-17

**Authors:** Nargish Parvin, Mohammad Aslam, Md Najib Alam, Tapas K. Mandal

**Affiliations:** 1 School of Mechanical Engineering, Yeungnam University, Gyeongsan 38541, Republic of Korea; nargish.parvin@gmail.com; 2 School of Chemical Engineering, Yeungnam University, Gyeongsan 38541, Republic of Korea; mohammadaslam13@gmail.com

**Keywords:** smart nanomaterials, targeted drug delivery, stimuli-responsive systems, regenerative medicine, pharmaceutical nanotechnology

## Abstract

The integration of smart nanomaterials into pharmaceutics has transformed approaches to disease diagnosis, targeted therapy, and tissue regeneration. These nanoscale materials exhibit unique features such as controlled responsiveness, biocompatibility, and precise site-specific action, offering new possibilities for personalized healthcare. This review provides a comprehensive overview of recent advances in the design and application of functional nanomaterials, including nanoparticle-based drug carriers, responsive hydrogels, and nanostructured scaffolds. Special focus is placed on stimuli-triggered systems that achieve controlled drug release and localized therapeutic effects. In addition, the review explores how these materials enhance diagnostic imaging and support tissue regeneration through adaptive and multifunctional designs. Importantly, this work uniquely integrates stimuli-responsive nanomaterials across therapeutic, imaging, and regenerative domains, providing a unified view of their biomedical potential. The challenges of clinical translation, large-scale synthesis, and regulatory approval are critically analyzed to outline future directions for research and real-world implementation. Overall, this review highlights the pivotal role of smart nanomaterials in advancing modern pharmaceutics toward more effective and patient-centered therapies.

## 1. Introduction

### 1.1. Overview of Nanomaterials in Pharmaceutics

Over the last three decades, nanomaterials have emerged as transformative tools in pharmaceutics, reshaping how we understand, design, and deliver therapeutic agents. Traditional drug formulations, while effective in many cases, often face several limitations, including poor solubility, low bioavailability, rapid systemic clearance, and off-target side effects. Nanomaterials provide a unique platform to overcome these barriers by leveraging their nanoscale size, tunable physicochemical properties, and functional versatility. By definition, nanomaterials are materials engineered at dimensions typically ranging from 1 to 100 nanometers. Within this scale, matter demonstrates novel optical, mechanical, magnetic, and biochemical properties that are distinct from their bulk counterparts, making them particularly well-suited for biomedical and pharmaceutical applications [1,2,3]. One of the most significant contributions of nanomaterials to pharmaceutics lies in the development of nanoparticle-based drug delivery systems. Liposomes, polymeric nanoparticles, dendrimers, micelles, and inorganic nanostructures have all been widely studied as drug carriers. Their small size facilitates enhanced permeability and retention (EPR) effect in tumor tissues, allowing for passive targeting of solid tumors. Beyond this, surface functionalization with ligands such as antibodies, peptides, or small molecules enables active targeting, ensuring that therapeutic agents accumulate specifically at diseased sites while sparing healthy tissues [3,4,5,6]. This dual advantage of passive and active targeting has made nanomaterials indispensable in advancing precision medicine. Furthermore, nanomaterials offer solutions to the long-standing challenge of poorly water-soluble drugs. Many promising therapeutic molecules fail during preclinical or clinical development due to poor solubility and low absorption. Nanocarriers, by encapsulating these drugs or modifying their physicochemical interactions, significantly enhance solubility and pharmacokinetics, ultimately improving clinical outcomes [7]. In addition, nanostructures can provide sustained or controlled drug release, maintaining therapeutic concentrations for longer durations while minimizing dosing frequency and adverse effects [8]. Beyond drug delivery, nanomaterials play a pivotal role in pharmaceutical diagnostics and theranostics. Quantum dots, iron oxide nanoparticles, and gold nanostructures, for instance, exhibit remarkable imaging capabilities due to their unique optical and magnetic properties. These nanoprobes allow early disease detection, real-time monitoring of therapeutic efficacy, and even simultaneous diagnosis and treatment in integrated theranostic systems [9]. This convergence of therapy and diagnostics exemplifies the broader potential of nanotechnology in reshaping pharmaceutics from a purely therapeutic discipline into a comprehensive healthcare strategy. In addition to synthetic nanomaterials, biomimetic and bioinspired nanostructures have recently attracted significant interest. For instance, cell membrane-coated nanoparticles harness the natural biological functions of cell surfaces, such as immune evasion or tissue homing, thereby enhancing therapeutic performance [10]. Similarly, protein and peptide-based nanostructures offer high biocompatibility and the ability to respond to specific biological cues. These innovations underscore the evolving integration of nanotechnology with biomolecular sciences. However, despite these advances, challenges remain in translating nanomaterials from laboratory research to clinical practice. Issues related to large-scale manufacturing, reproducibility, long-term stability, and potential toxicity must be carefully addressed. Moreover, regulatory frameworks specific to nanomedicine are still evolving, creating uncertainties for commercialization [11]. Nevertheless, the body of evidence supporting nanomaterial-based pharmaceutics continues to expand, and their impact on drug development and healthcare delivery is undeniable. In summary, nanomaterials have redefined the pharmaceutical landscape by enabling enhanced drug delivery, improved diagnostic accuracy, and integrated theranostic systems. They offer the potential to address unmet medical needs while paving the way toward personalized and precision medicine. The following sections of this review focus on a more specialized class of these materials, smart nanomaterials that build upon the fundamental principles of nanotechnology to introduce responsiveness, adaptability, and multifunctionality to modern pharmaceutics. Unlike previous reviews that primarily focus on individual applications such as drug delivery or diagnostic imaging, this review provides an integrated perspective that connects three major biomedical areas as therapeutics, imaging, and tissue regeneration, within a unified framework of smart nanomaterials. The manuscript not only summarizes current progress but also emphasizes the translational barriers that influence clinical adoption, including large-scale production, safety validation, and regulatory approval. By combining technical insights with a discussion of real-world challenges, this review aims to bridge the gap between laboratory innovation and clinical implementation, offering a broader and more practical understanding of how smart nanomaterials are shaping next-generation pharmaceutics.

In this review, the term nanomaterials refers broadly to materials engineered at the nanoscale (1–100 nm) that exhibit unique physicochemical properties relevant to biomedical applications. The term nanocarriers is used specifically for nanoscale systems designed to transport and release therapeutic or diagnostic agents, such as liposomes, micelles, or polymeric nanoparticles. Nanoplatforms, in contrast, describe multifunctional or composite systems that integrate multiple components such as targeting ligands, imaging agents, and stimuli-responsive elements within a single construct. These distinctions are maintained throughout the manuscript to ensure conceptual clarity and consistent terminology.

### 1.2. Emergence of Smart Nanomaterials

While conventional nanomaterials have already significantly advanced pharmaceutics, the field is now witnessing a new era with the development of smart nanomaterials engineered systems that can sense, respond, and adapt to specific biological or external stimuli. Unlike traditional drug carriers that passively release their cargo over time, smart nanomaterials exhibit dynamic properties, enabling them to perform sophisticated tasks such as site-specific drug release, multi-modal imaging, or even regenerative functions. This adaptability makes them particularly attractive in addressing complex diseases like cancer, neurodegeneration, and chronic inflammatory disorders [12]. The emergence of smart nanomaterials is closely tied to advances in materials science, biotechnology, and nanofabrication. Stimuli-responsive systems are at the core of this innovation. These nanomaterials can respond to a variety of triggers such as pH, temperature, light, magnetic fields, enzymes, or redox gradients that are often uniquely altered in pathological environments [13]. For example, tumor tissues often exhibit lower pH and higher reactive oxygen species compared to normal tissues. Smart nanoparticles engineered to release drugs only under these conditions ensure that therapeutic action is highly localized, thereby improving efficacy while minimizing systemic toxicity [14]. Among the many categories of smart nanomaterials, polymeric nanocarriers with pH or temperature-sensitive linkages are widely studied for controlled release applications. Similarly, light-responsive nanomaterials, including gold nanorods and carbon-based structures, enable precise spatial and temporal control over drug activation through photothermal or photodynamic effects [15]. Magnetic nanoparticles, on the other hand, allow for externally guided delivery and on-demand release when exposed to alternating magnetic fields [16]. These examples highlight the breadth of strategies being employed to introduce intelligence into nanomaterials. Beyond drug delivery, smart nanomaterials are increasingly being integrated into regenerative medicine. Nanostructured hydrogels, for instance, can mimic the dynamic mechanical and biochemical environment of native tissues, promoting cell adhesion, proliferation, and differentiation. By incorporating stimuli-responsive components, these scaffolds can provide controlled release of growth factors or respond to cell-secreted signals, thereby creating a feedback loop that supports tissue regeneration [17,18]. Similarly, bioactive nanostructures functionalized with peptides or extracellular matrix proteins can guide stem cell fate and accelerate wound healing. Another transformative area is theranostics, where smart nanomaterials enable simultaneous diagnosis and therapy. Multifunctional nanoplatforms equipped with imaging probes and therapeutic agents can identify diseased sites, track treatment progress, and deliver targeted interventions in real time [19]. Such integrated systems are particularly relevant for oncology, where early detection and precise treatment are critical to patient outcomes. Despite their promise, smart nanomaterials face unique challenges. Their increased complexity often makes synthesis and scale-up more difficult compared to conventional nanomaterials. Additionally, ensuring reproducibility, biocompatibility, and long-term safety requires rigorous testing. Regulatory pathways for such adaptive systems are also less defined, raising hurdles for clinical translation [20]. Nonetheless, the trajectory of research indicates that smart nanomaterials will play a central role in next-generation pharmaceutics, offering solutions that go beyond static drug delivery toward dynamic, patient-tailored therapies.

### 1.3. Objectives and Scope of This Review

Given the rapid evolution and strong potential of smart nanomaterials in pharmaceutics, this review aims to provide a comprehensive and forward-looking perspective on the field. While numerous reviews exist on conventional nanomaterials and their applications in drug delivery, fewer have specifically focused on the unique category of “smart” nanomaterials, which represent the next frontier in biomedical innovation. Thus, the objective of this manuscript is not merely to summarize existing knowledge but to critically analyze emerging trends, highlight innovative designs, and discuss the translational challenges associated with these advanced systems. The scope of this review spans several key areas. First, we examine the fundamental design principles of smart nanomaterials, including their structural features, stimuli-responsive mechanisms, and functional versatility. Special emphasis is placed on how these materials differ from traditional nanocarriers in their ability to interact with biological environments dynamically. Second, we explore their applications across major domains of pharmaceutics, ranging from targeted drug delivery and controlled release systems to diagnostic imaging, theranostics, and regenerative medicine [6,11,21]. By integrating insights from these diverse applications, we aim to illustrate the cross-disciplinary impact of smart nanotechnology. In addition to surveying current progress, this review also addresses critical challenges and limitations. Issues such as large-scale synthesis, reproducibility, long-term biocompatibility, and regulatory uncertainties are thoroughly discussed to provide a balanced perspective. Equally important, we consider the ethical implications of deploying such advanced technologies in clinical settings, including patient safety, data privacy in diagnostic applications, and equitable access to personalized medicine [22]. Finally, this review seeks to identify future directions for research and development. Topics such as the integration of smart nanomaterials with artificial intelligence, 3D bioprinting, and bioelectronics are introduced as emerging trends that may redefine pharmaceutics in the coming decades. By highlighting these opportunities, the review aims to serve as both a resource for current researchers and a roadmap for future innovations.

In summary, this review sets out to achieve three primary goals:

1. To provide a detailed overview of the design and functionality of smart nanomaterials in pharmaceutics.
2. To critically evaluate their applications across drug delivery, diagnostics, and regenerative medicine.
3. To discuss translational challenges and propose future perspectives for clinical adoption.

Through this structured approach, we intend to underline the transformative role of smart nanomaterials in shaping the future of pharmaceutics, bridging the gap between laboratory research and patient-centered healthcare. In addition to summarizing recent advancements, this review critically evaluates the comparative performance of different smart nanomaterials and their translation from experimental models to clinical settings. While many laboratory studies demonstrate promising results in targeted delivery and controlled release, only a limited number of nanoplatforms have successfully advanced to clinical trials due to issues related to large-scale synthesis, reproducibility, and long-term safety. Moreover, disparities between preclinical and human outcomes often arise from differences in physiological environments and disease heterogeneity. Therefore, the review emphasizes the importance of correlating in vitro performance with in vivo validation, considering pharmacokinetics, immune response, and regulatory compliance as key factors. By integrating these analytical perspectives, the manuscript aims to provide readers with a balanced understanding of both the technological progress and the current limitations that influence the real-world applicability of smart nanomaterials in pharmaceutics.

## 2. Design and Properties of Smart Nanomaterials

### 2.1. Stimuli-Responsive Behavior

Building upon the above critical evaluation, the following section explores the functional mechanisms of smart nanomaterials, focusing on their stimuli-responsive behavior and biomedical applications.

#### 2.1.1. pH Responsive Nanomaterials

The physiological environment of the human body is characterized by diverse pH gradients, which serve as a natural trigger for smart nanomaterials. For example, the bloodstream typically maintains a near neutral pH of ~7.4, while tumor microenvironments and inflamed tissues often exhibit acidic conditions ranging from pH 6.0–6.8. Endosomal and lysosomal compartments within cells are even more acidic (pH 4.5–6.0). Exploiting these differences, pH-responsive nanomaterials are designed to remain stable at physiological pH but undergo structural or chemical transformations in acidic environments, resulting in targeted drug release [23,24,25]. One common design involves nanocarriers with acid-labile bonds such as hydrazone, acetal, imine, or cis aconityl linkages. These bonds remain intact at neutral pH but are cleaved under acidic conditions, leading to rapid drug release specifically within diseased sites. For instance, polymeric micelles with hydrazone linkages between the hydrophobic core and therapeutic molecules have been successfully used for selective release in tumor tissues [26]. Similarly, liposomes coated with pH-sensitive polymers destabilize in acidic compartments, enhancing intracellular delivery of encapsulated drugs [27]. Another strategy employs charge conversional systems. Certain polymers or peptides undergo a charge shift when exposed to acidic pH. For example, zwitterionic materials can switch to a positively charged state in acidic environments, improving cellular uptake due to enhanced electrostatic interaction with negatively charged cell membranes [28]. This approach not only facilitates drug delivery but also promotes deeper penetration into tumor tissues. pH-responsive nanomaterials have also been integrated into multifunctional systems that combine therapy with diagnostics (theranostics). For example, pH-sensitive polymer-coated gold nanoparticles enable drug release and simultaneous imaging through surface plasmon resonance. Similarly, quantum dots and iron oxide nanoparticles coated with acid-labile polymers act as dual-purpose platforms for imaging and therapy [29]. Despite their promise, pH-responsive systems face challenges in achieving precise control, as physiological pH differences can be subtle and heterogeneous across tissues. In addition, premature drug release due to systemic acidosis in certain conditions (e.g., sepsis) must be minimized. Future research is focusing on integrating pH responsiveness with other stimuli, such as redox or enzymatic triggers, to create dual or multi-stimuli-responsive platforms with improved specificity. For example, a study demonstrated that doxorubicin-loaded polymeric micelles containing hydrazone linkages achieved over 80% drug release within 6 h at pH 5.5, compared to less than 10% at physiological pH 7.4 [30]. In vivo testing in mice bearing MCF-7 tumors showed a 68% reduction in tumor volume after 14 days, confirming the therapeutic benefit of acid-triggered release. Such quantitative outcomes clearly validate the precision and efficacy of pH-responsive nanocarriers for cancer therapy.

#### 2.1.2. Temperature Responsive Systems

Temperature is another key physiological parameter that varies in diseased versus normal tissues. Localized hyperthermia is often observed in tumors, inflamed regions, or infected sites. In addition, external thermal triggers, such as infrared radiation or magnetic fields, can be applied to elevate local temperatures. Temperature-responsive nanomaterials harness these variations to achieve controlled drug release, tunable sol–gel transitions, or triggered structural changes [16]. The most widely studied systems are thermo-responsive polymers, particularly those exhibiting a lower critical solution temperature (LCST). Polymers such as poly(N-isopropylacrylamide) (PNIPAM) remain hydrated and soluble below their LCST (~32 °C) but undergo phase transition to a hydrophobic, collapsed state above LCST. This switch can be exploited to release encapsulated drugs upon exposure to hyperthermic conditions [31]. By tuning the LCST through copolymerization with hydrophilic or hydrophobic monomers, the system can be adapted for physiological or disease-specific applications. Hydrogels represent another class of temperature-responsive materials. Injectable hydrogels composed of block copolymers (e.g., Pluronic F127, PEG PCL PEG) exhibit sol to gel transitions at body temperature, enabling minimally invasive administration and localized drug depot formation [32]. These materials are particularly attractive for cancer therapy and tissue engineering, where controlled release and scaffold formation are essential.

Additionally, temperature-sensitive liposomes (TSLs) have been developed to release their cargo in response to mild hyperthermia (40–42 °C). These formulations are often combined with external energy sources such as ultrasound or near infrared (NIR) irradiation to precisely regulate drug release at target sites [33]. A well-studied example is ThermoDox^®^, a lysolipid-based TSL under clinical trials for cancer treatment. Despite extensive research, temperature-responsive systems face certain limitations. One challenge is the difficulty of achieving uniform and sustained hyperthermia in deep tissues without damaging surrounding healthy structures. Another is ensuring that the thermal trigger induces consistent and reproducible responses across different patients. Integrating temperature responsiveness with magnetic nanoparticles or NIR absorbing agents has been proposed as a solution, offering non-invasive and localized heating for precise therapeutic action. In a preclinical evaluation, a thermosensitive liposomal formulation similar to ThermoDox^®^ released nearly 70% of its doxorubicin payload when exposed to mild hyperthermia (42 °C) for 10 min, while maintaining below 5% release at 37 °C [33,34]. When combined with localized heat treatment, tumor regression in mouse xenografts reached 75%, compared with only 30% for conventional liposomes. These results demonstrate the effectiveness of temperature-triggered systems in achieving spatiotemporal control of drug delivery.

#### 2.1.3. Redox Responsive Nanoplatforms

Redox potential is a critical biochemical feature of cellular microenvironments. While extracellular spaces typically maintain oxidizing conditions, intracellular compartments such as the cytosol and mitochondria exhibit reducing conditions due to high concentrations of glutathione (GSH), often reaching 2–10 mM. In contrast, extracellular GSH levels are typically in the micromolar range. Moreover, many pathological conditions, including cancer and inflammatory diseases, are associated with disrupted redox homeostasis and elevated reactive oxygen species (ROS). These gradients provide opportunities for redox-responsive nanomaterials that release drugs selectively within target cells [35]. A common design strategy involves nanocarriers incorporating disulfide bonds. Disulfide linkages are stable under extracellular conditions but are cleaved in the presence of high intracellular GSH, resulting in triggered release. Polymeric micelles, dendrimers, and hydrogels crosslinked via disulfide bonds have been extensively studied for intracellular delivery of chemotherapeutics [36]. For example, doxorubicin-loaded disulfide-crosslinked micelles demonstrate enhanced release in cancer cells while minimizing systemic leakage. Another approach involves ROS responsive systems. Polymers containing thioketal, boronic ester, or peroxalate linkages degrade upon exposure to elevated ROS, releasing encapsulated drugs specifically at inflamed or tumor sites [37]. ROS-sensitive nanoplatforms are particularly relevant for inflammatory diseases such as arthritis, where localized oxidative stress provides a natural trigger.

Redox-responsive systems are also being integrated with gene delivery platforms. Nucleic acid-based therapeutics, such as siRNA or CRISPR components, are notoriously unstable in circulation and require efficient intracellular release. Redox cleavable carriers ensure that genetic cargo is protected in systemic circulation but released upon cellular entry, improving gene therapy efficiency [38,39]. Despite their advantages, challenges remain in precisely controlling the degree of redox sensitivity. Excessive sensitivity may lead to premature degradation, whereas insufficient responsiveness reduces therapeutic efficiency. Furthermore, variations in intracellular GSH or ROS levels between patients and disease types complicate clinical translation. Current trends focus on dual responsive nanoplatforms combining redox sensitivity with pH or enzymatic responsiveness to improve selectivity and minimize off-target effects. A redox-sensitive micellar system was developed and showed 85% drug release within 8 h in a 10 mM glutathione environment, mimicking intracellular conditions, but only 12% release under extracellular oxidative conditions [40,41]. In vivo, the formulation achieved a 3.5-fold increase in tumor growth inhibition compared to non-responsive controls, indicating the strong therapeutic advantage of redox-cleavable nanocarriers in targeting intracellular pathways.

#### 2.1.4. Light and Magnetic Field Responsive Materials

External physical stimuli such as light and magnetic fields offer unique opportunities for non-invasive, spatiotemporally controlled drug release. These exogenous triggers provide high precision, enabling site-specific therapy while minimizing systemic exposure. Light-responsive nanomaterials are particularly attractive due to their ability to utilize near-infrared (NIR) light, which penetrates tissues up to several centimeters with minimal damage. Gold nanoparticles, nanorods, and nanoshells exhibit strong surface plasmon resonance, converting absorbed NIR light into heat (photothermal effect), thereby inducing localized hyperthermia for cancer ablation [42]. Alternatively, light-sensitive linkages such as o-nitrobenzyl esters can be incorporated into polymeric carriers, allowing drug release upon photolysis [43]. Light-responsive hydrogels have also been designed to undergo gel–sol transitions upon irradiation, offering potential for on-demand drug delivery or injectable implants. Magnetic responsive nanomaterials, particularly those based on iron oxide nanoparticles (IONPs), have gained attention for their dual role in therapy and diagnostics. Under an alternating magnetic field, IONPs generate localized heat through magnetic hyperthermia, which can trigger drug release from thermosensitive carriers or directly ablate tumor tissues [16]. Moreover, IONPs serve as contrast agents in magnetic resonance imaging (MRI), enabling theranostic applications. Magnetic guidance is another valuable feature, where drug-loaded IONPs can be directed to specific tissues using external magnets, thereby improving targeting efficiency. Integration of light and magnetic responsiveness into multifunctional nanoplatforms has created opportunities for synergistic therapies. For example, nanocomposites combining gold nanorods and IONPs allow simultaneous photothermal therapy, magnetic hyperthermia, and MRI tracking. Such systems are particularly promising for cancer treatment, where multimodal action can overcome tumor heterogeneity and resistance [44]. Despite these advancements, limitations remain. Light penetration depth, particularly in deep tissues, is restricted, limiting the applicability of phototherapies. Magnetic hyperthermia, while promising, requires precise control of field strength and frequency to avoid tissue damage. Ongoing research is addressing these issues by developing second window NIR materials, magnetic nanocomposites with enhanced heating efficiency, and implantable devices that combine multiple stimuli for precise therapeutic action [45]. Table 1 highlights the diverse range of stimuli-responsive nanomaterials that have emerged as promising platforms in modern biomedical applications. These smart materials are engineered to respond to specific internal triggers, such as pH, redox conditions, and enzymatic activity, or external stimuli like light, temperature, ultrasound, and magnetic fields. Such responsiveness enables controlled drug release, targeted delivery, and improved therapeutic precision while minimizing off-target effects. For example, pH or redox-sensitive systems exploit the tumor microenvironment for selective drug release, whereas light and magnetically responsive nanomaterials offer spatiotemporal control over therapeutic action. Despite these advantages, challenges remain in achieving reproducibility, stability, biocompatibility, and scalability, which are essential for clinical translation. Overall, stimuli-responsive nanomaterials represent a critical step toward next-generation precision nanomedicine, but addressing their limitations will be key for their successful deployment in real-world biomedical settings. In a representative study, gold nanorods exposed to near infrared (808 nm) irradiation generated localized temperatures exceeding 45 °C, resulting in over 90% ablation of tumor cells in vitro and a 65% reduction in tumor volume in vivo [46,47]. Similarly, iron oxide nanoparticles used for magnetic hyperthermia achieved approximately 80% tumor regression in mouse models when subjected to alternating magnetic fields at 200 kHz. These findings underscore how photothermal and magnetic triggers provide precise and quantifiable control of therapeutic outcomes.

### 2.2. Biocompatibility and Biodegradability Considerations

Biocompatibility and biodegradability are two of the most crucial parameters that determine the clinical success of smart nanomaterials in pharmaceutics. While stimuli-responsive and multifunctional properties offer therapeutic advantages, their safety profile dictates whether they can be translated from laboratory models into approved medical applications. In particular, nanomaterials interact intimately with biological systems blood, cells, tissues, and organs, making it essential to evaluate their compatibility and degradation behavior in vivo [48]. Biocompatibility and biodegradability represent two distinct but interrelated aspects of nanomaterial safety. Short-term biocompatibility focuses on the immediate physiological interactions of nanomaterials, including their cytotoxicity toward mammalian cells, hemolytic potential in blood, and the degree of inflammatory or immune activation following administration. These responses are usually assessed through in vitro cell viability assays, hemocompatibility tests, and cytokine analysis in animal models. In contrast, long-term biodegradability refers to the breakdown, clearance, and accumulation behavior of nanomaterials over extended periods. Biodegradable polymers such as PLGA and PCL degrade into metabolizable byproducts, while inorganic nanomaterials often show slower clearance. For example, although ultrasmall gold nanoparticles (<5 nm) can undergo renal excretion, most therapeutic gold nanoparticles typically exceed this size and are retained within the reticuloendothelial system, especially in the liver and spleen. Such accumulation underscores potential long-term safety concerns, including oxidative stress or chronic inflammation. Therefore, designing nanomaterials with controlled degradation profiles and predictable clearance pathways remains essential to achieving both short-term safety and long-term biocompatibility.

**Figure 1:**
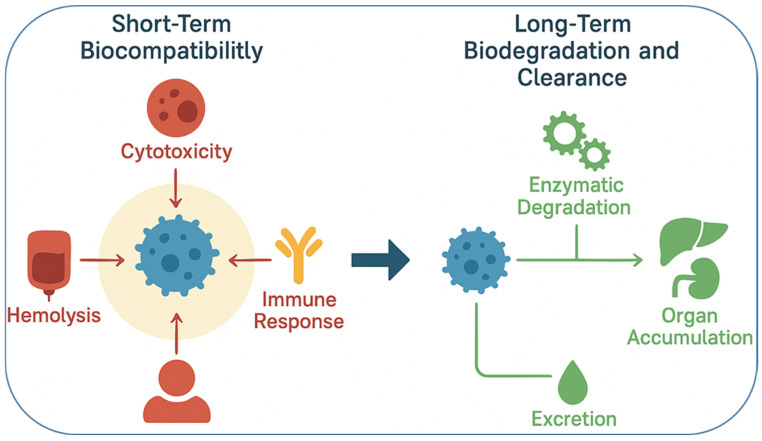
Schematic representation of biocompatibility and biodegradability considerations in smart nanomaterials. Biocompatibility assessment involves evaluating cytotoxicity, immune activation, and hemolysis to ensure safety in biological systems. Biodegradability focuses on designing nanocarriers with cleavable linkages that degrade into non-toxic byproducts, enabling safe clearance and reducing long-term accumulation risks.

Biocompatibility refers to the ability of a nanomaterial to perform its intended function without eliciting undesirable local or systemic effects. For drug delivery applications, this means that nanocarriers should not induce cytotoxicity, hemolysis, immune activation, or inflammatory responses beyond acceptable limits [49]. Traditional nanocarriers such as liposomes and polymeric micelles are generally well tolerated, as they are composed of biomimetic or FDA-approved polymers like polyethylene glycol (PEG) and poly(lactic co-glycolic acid) (PLGA). However, the incorporation of advanced functional groups, metallic nanostructures, or stimuli-sensitive linkages can introduce new toxicity risks. For example, certain cationic polymers exhibit excellent nucleic acid binding but can disrupt cellular membranes, leading to dose-dependent cytotoxicity [50]. Similarly, inorganic nanoparticles such as quantum dots may release toxic heavy metals if not properly coated or stabilized [51]. To assess biocompatibility, a systematic evaluation is necessary, including in vitro assays (cell viability, hemolysis, oxidative stress, and complement activation) and in vivo studies (organ distribution, immunogenicity, and long-term clearance). The interaction of nanomaterials with the immune system is particularly critical. For instance, opsonization by plasma proteins often leads to rapid clearance by the reticuloendothelial system (RES), limiting circulation time and therapeutic efficacy [52]. PEGylation, surface coating with polysaccharides, or cloaking with natural cell membranes are among the strategies employed to reduce immune recognition and improve biocompatibility [53]. Biodegradability is equally important, as it ensures that nanomaterials are eventually broken down into non-toxic byproducts that can be safely eliminated from the body. Biodegradable systems minimize long-term accumulation in tissues, which is a major concern for inorganic nanostructures that are not readily metabolized. Polymers such as PLGA, polylactic acid (PLA), and polycaprolactone (PCL) degrade through hydrolysis into naturally metabolized monomers, making them widely used in clinical applications [54]. Similarly, natural polymers like chitosan, alginate, gelatin, and hyaluronic acid are inherently biodegradable and biocompatible, though they may require chemical modification for improved stability [55]. Inorganic nanomaterials, including gold, iron oxide, and silica nanoparticles, pose more challenges due to limited biodegradability. However, progress has been made in engineering these systems for clearance. For instance, ultrasmall (<5 nm) gold nanoparticles can undergo renal clearance, while biodegradable mesoporous silica nanoparticles dissolve gradually into non-toxic silicic acid under physiological conditions [56]. Surface modifications with biodegradable coatings also improve clearance. An additional consideration is the degradation kinetics of smart nanomaterials, which must align with therapeutic needs. For example, rapid degradation may compromise drug delivery efficiency, whereas excessively slow degradation can lead to bioaccumulation and chronic toxicity [57]. Therefore, designing systems with tunable degradation profiles controlled by polymer composition, crosslinking density, or environmental triggers is an area of active research. It is also important to recognize that biocompatibility and biodegradability are context-dependent. A nanomaterial that is safe for short-term drug delivery may not be suitable for long-term implants. Likewise, degradation byproducts may have unexpected biological effects, such as altering pH or triggering inflammation [58]. Thus, a balance must be achieved between functional performance and safety. Regulatory agencies such as the FDA and EMA increasingly require comprehensive safety assessments specific to nanomaterials, including pharmacokinetics, biodistribution, immunotoxicity, and genotoxicity studies [59]. These requirements emphasize that biocompatibility and biodegradability are not merely academic concerns but central to the clinical adoption of smart nanomaterials. In summary, the integration of smart nanomaterials into pharmaceutics must be accompanied by rigorous evaluation of their biocompatibility and biodegradability. Strategies such as using natural or FDA-approved polymers, engineering biodegradable linkages, minimizing toxic components, and designing clearance pathways, are vital to ensuring safety. Ultimately, achieving an optimal balance between functionality and safety will determine the success of smart nanomaterials in clinical translation. Figure 1 highlights the dual importance of biocompatibility and biodegradability in the rational design of smart nanomaterials for biomedical applications. Biocompatibility ensures that nanomaterials do not trigger adverse cellular or systemic responses, such as cytotoxicity, inflammation, or immune activation, which are critical for their safe interaction with living systems. On the other hand, biodegradability addresses the long-term safety of these materials by enabling their breakdown into harmless, excretable byproducts, thereby preventing unwanted accumulation in tissues and organs. Together, these properties form the foundation for clinical translation, as they directly influence therapeutic efficacy, patient safety, and regulatory acceptance of nanotechnology-based interventions. Table 2 provides a comparative overview of the biodegradation and clearance pathways of key nanomaterial classes, emphasizing how their physicochemical composition directly influences biological fate and safety. Polymeric nanomaterials such as PLGA, PLA, and PCL degrade primarily through hydrolysis into metabolizable monomers, offering predictable clearance and well-established clinical compatibility. Lipid-based carriers are enzymatically degraded by lipases and phospholipases, producing biocompatible metabolites that are efficiently processed through hepatic pathways, explaining their frequent approval for pharmaceutical use. In contrast, metallic nanoparticles exhibit slower or incomplete degradation, with most particles above 10 nm accumulating in the liver and spleen due to uptake by the reticuloendothelial system. Hybrid nanomaterials combine these behaviors, showing complex degradation kinetics influenced by both organic and inorganic components. Understanding these mechanisms is crucial for optimizing nanocarrier design, as the rate and route of clearance determine long-term biocompatibility, toxicity risk, and clinical translatability.

### 2.3. Surface Modification and Functionalization Strategies

The performance of smart nanomaterials in pharmaceutics is heavily influenced by their surface properties. While the core composition determines bulk properties such as size, stability, and degradation, the surface interface dictates how nanomaterials interact with biological systems. Surface modification and functionalization strategies, therefore, represent a cornerstone of nanomaterial design, enabling enhanced stability, targeted delivery, immune evasion, and multifunctionality [60]. One of the most widely employed strategies is polymer coating, particularly with polyethylene glycol (PEG). PEGylation creates a hydrophilic corona that reduces protein adsorption (opsonization), prolongs circulation half-life, and minimizes immune recognition [61]. For instance, PEGylated liposomes such as Doxil^®^ have demonstrated improved pharmacokinetics and reduced cardiotoxicity compared to free doxorubicin. Beyond PEG, zwitterionic polymers and polysaccharides (e.g., dextran, hyaluronic acid, chitosan) are increasingly explored as alternatives, offering similar stealth properties with reduced concerns about PEG immunogenicity [62]. Ligand functionalization is another critical approach to achieve active targeting. By conjugating targeting moieties such as antibodies, peptides, aptamers, or small molecules, nanocarriers can recognize and bind to specific receptors overexpressed on diseased cells. For example, folate-functionalized nanoparticles selectively target cancer cells that overexpress folate receptors, while RGD peptides enhance binding to integrin-rich tumor vasculature [63]. In regenerative medicine, ligands that mimic extracellular matrix (ECM) proteins guide cell adhesion and differentiation [64]. The choice of targeting ligand depends on the disease context and requires optimization to balance binding affinity with systemic circulation properties. Stimuli-sensitive functionalization represents an advanced strategy that aligns with the responsive nature of smart nanomaterials. For instance, surfaces can be engineered with acid-labile linkers that detach under acidic conditions, or with enzyme-cleavable peptides that release therapeutic payloads upon encountering specific enzymes in diseased tissues [65]. Such functionalization not only improves specificity but also integrates diagnostic or therapeutic triggers directly into the nanomaterial surface. Nanomaterials are also frequently engineered with multifunctional surfaces for theranostic applications. For instance, gold nanoparticles coated with tumor-targeting ligands and fluorescent dyes enable simultaneous imaging and therapy. Similarly, iron oxide nanoparticles functionalized with PEG, drugs, and targeting ligands combine MRI contrast with therapeutic delivery [66]. Layer-by-layer assembly of polyelectrolytes, lipids, and bioactive molecules further enables the construction of complex multifunctional coatings [67]. While PEGylation has long been regarded as an effective method for improving nanoparticle stability, extending circulation time, and minimizing recognition by the reticuloendothelial system, recent reports have identified the development of anti-PEG antibodies in some patients. These antibodies can accelerate blood clearance upon repeated administration, reducing therapeutic efficacy and, in some cases, eliciting mild immune responses. This phenomenon, known as the accelerated blood clearance (ABC) effect, has prompted growing attention toward alternative “stealth” coatings that maintain similar benefits without triggering immune recognition. Natural polysaccharides such as dextran, hyaluronic acid, and chitosan derivatives, along with synthetic zwitterionic polymers, have shown promise in reducing opsonization while avoiding anti-PEG immune responses. Therefore, PEGylation continues to play a central role in nanomedicine, but careful evaluation of immunogenicity and the exploration of alternative surface coatings are increasingly important for achieving long-term biocompatibility and consistent therapeutic performance.

Another innovative trend is the development of biomimetic coatings. By cloaking nanoparticles with natural cell membranes derived from red blood cells, platelets, or cancer cells, researchers can create systems that inherit the biological functionalities of these membranes. Such coatings enable immune evasion, prolonged circulation, and even homotypic targeting in the case of cancer cell membrane cloaked nanoparticles [68]. This strategy bridges nanotechnology with biology, creating highly sophisticated drug delivery systems. Surface modification also plays a crucial role in controlling pharmacokinetics and biodistribution. For instance, hydrophilic coatings improve circulation time, while hydrophobic modifications enhance membrane penetration. Similarly, positively charged surfaces facilitate cellular uptake but risk cytotoxicity, whereas neutral or zwitterionic surfaces minimize immune clearance but may reduce uptake efficiency [69]. Balancing these competing factors requires rational design informed by disease-specific needs. From a technical perspective, several chemical strategies are employed for functionalization, including carbodiimide chemistry, thiol gold interactions, click chemistry, and biotin–streptavidin linkages [70]. These methods enable precise and stable attachment of ligands while preserving bioactivity. Advances in site-specific conjugation and bio-orthogonal chemistry further improve reproducibility and scalability, both essential for clinical translation. Challenges remain in ensuring that functionalized surfaces maintain stability in vivo. The “protein corona” formed upon contact with biological fluids can mask surface ligands, reducing targeting efficiency. Approaches such as corona-resistant coatings, pre-adsorption of specific proteins, or dynamic exchange-resistant materials are being explored to address this issue [71]. Beyond improving circulation stability and targeted delivery, surface functionalization plays a pivotal role in enhancing both imaging accuracy and tissue regeneration. In imaging applications, the conjugation of targeting ligands or antibodies to iron oxide nanoparticles markedly improves MRI precision by enabling selective accumulation in diseased tissues. For instance, folate or RGD functionalized superparamagnetic iron oxide nanoparticles (SPIONs) have demonstrated significantly higher T_2_ contrast enhancement in tumor imaging compared to unmodified particles, confirming the importance of active surface targeting in diagnostic accuracy. Similarly, PEGylation combined with fluorescent or magnetic labels improves nanoparticle stability in circulation, leading to sharper imaging resolution and reduced background noise. In regenerative medicine, functionalization with ECM-derived peptides such as RGD or collagen mimetic sequences facilitates cell adhesion, proliferation, and lineage-specific differentiation. For example, hydrogel scaffolds decorated with these bioactive motifs promote osteogenic or neurogenic differentiation by mimicking native tissue microenvironments. Such biomimetic modifications bridge the gap between material functionality and biological performance, demonstrating that surface engineering not only guides therapeutic targeting but also directly supports tissue regeneration and repair.

In conclusion, surface modification and functionalization are indispensable strategies in the design of smart nanomaterials. By fine-tuning the surface, researchers can achieve immune evasion, targeted delivery, controlled release, and multifunctionality. As nanomedicine advances toward clinical adoption, the ability to engineer surfaces with precision and stability will be central to translating laboratory innovations into practical pharmaceutical solutions.

## 3. Smart Nanomaterials for Targeted Drug Delivery

### 3.1. Active Targeting Approaches

Active targeting represents one of the most promising strategies in nanomedicine, as it allows drug-loaded nanomaterials to be directed specifically toward diseased tissues or cells by exploiting molecular recognition mechanisms. Unlike passive targeting, which relies on the enhanced permeability and retention (EPR) effect, active targeting involves functionalizing nanocarriers with ligands, antibodies, peptides, aptamers, or small molecules that selectively bind to receptors or biomolecules expressed at high levels on pathological cells. This approach not only increases therapeutic efficacy but also reduces off-target toxicity, which remains one of the central challenges in conventional drug delivery systems. Recent advances in nanotechnology have made it possible to design multifunctional nanoplatforms capable of combining high selectivity with controlled release features, thereby improving the clinical translation potential of nanomedicine [11,72]. Overall, active targeting offers an efficient pathway to overcome biological barriers and enhance precision in therapy.

#### 3.1.1. Ligand Receptor Interactions

Ligand receptor interactions form the cornerstone of active targeting in smart nanomaterials, where ligands such as folic acid, transferrin, peptides, aptamers, and small molecules are conjugated to the nanocarrier surface. These ligands recognize overexpressed receptors on cancer cells, inflamed tissues, or other pathological sites, allowing the nanocarriers to selectively bind and internalize through receptor-mediated endocytosis. For instance, folic acid functionalized nanoparticles are widely studied because folate receptors are highly expressed in many tumor cells but are limited in normal tissues. This selective binding enhances drug accumulation within cancerous tissue while reducing systemic toxicity, an essential advantage for chemotherapeutics with narrow therapeutic windows [73]. Such mechanisms highlight how receptor overexpression can be exploited for disease-selective drug delivery. In addition to folic acid, transferrin has been extensively utilized as a targeting ligand due to the elevated expression of transferrin receptors in rapidly proliferating cancer cells. Transferrin-conjugated nanocarriers have demonstrated improved cellular uptake and intracellular trafficking compared to unmodified nanoparticles. Similarly, RGD (arginine–glycine–aspartic acid) peptides target integrins, which are upregulated in tumor angiogenesis, making them valuable tools for both therapy and imaging applications. These examples demonstrate the adaptability of ligand–receptor interactions for various disease contexts, enabling personalized treatment strategies based on receptor expression profiles [74]. The adaptability of ligands to different molecular targets suggests their versatility across multiple pathological indications. Moreover, aptamers, which are short single-stranded DNA or RNA molecules with high affinity for specific targets, have emerged as promising alternatives to antibodies. Aptamer-functionalized nanocarriers offer high binding specificity and biocompatibility while avoiding immunogenicity issues associated with proteins. Aptamer-mediated targeting of prostate-specific membrane antigen (PSMA), for instance, has shown remarkable selectivity in prostate cancer therapy. These nanosystems illustrate how ligand–receptor interactions are not limited to natural ligands but can also include synthetic biomolecules that expand the scope of targetable receptors [4]. The use of aptamers emphasizes the growing intersection of nanotechnology with nucleic acid engineering, broadening the horizon of active targeting. Despite the advantages, ligand–receptor targeting faces challenges such as heterogeneous receptor expression, ligand density optimization, and off-target binding. Overexpression of receptors may vary between patients or even within tumors, which can reduce therapeutic consistency. In addition, excessive ligand density can induce steric hindrance or recognition by the immune system, leading to premature clearance. Therefore, fine-tuning ligand presentation on nanocarrier surfaces is crucial to maximize targeting efficiency without compromising stealth properties [75]. Conclusively, ligand–receptor targeting is a powerful yet dynamic approach that must be optimized for each disease environment to achieve predictable and reproducible clinical outcomes.

#### 3.1.2. Antibody Conjugated Nanocarriers

Antibody-conjugated nanocarriers represent another major class of active targeting systems, capitalizing on the high specificity of monoclonal antibodies (mAbs) for antigens overexpressed in diseased tissues. Monoclonal antibodies have been widely used in cancer therapy, diagnostics, and immunotherapy, and their conjugation with nanocarriers enables dual benefits: the inherent therapeutic effect of the antibody and its targeting ability. For example, trastuzumab conjugated nanoparticles target HER2 receptors in breast cancer, combining the therapeutic effects of trastuzumab with the enhanced delivery of cytotoxic drugs encapsulated in the nanocarrier. This synergistic mechanism not only improves therapeutic efficacy but also reduces systemic exposure of the drug [76]. Such antibody-based targeting is particularly valuable for cancers with well-characterized biomarkers. Antibody–nanocarrier conjugates can be fabricated using covalent linkages, non-covalent interactions, or click chemistry-based methods. The stability of the conjugation method determines the circulation time, targeting efficiency, and drug release profile. Advances in site-specific conjugation techniques have allowed antibodies to be oriented correctly on the nanocarrier surface, ensuring maximum binding to their corresponding antigens. Moreover, antibody fragments such as Fab, scFv, and nanobodies have been investigated to reduce the size and immunogenicity of full-length antibodies while retaining binding specificity. These smaller constructs improve tumor penetration and minimize clearance by the reticuloendothelial system [77]. The adaptability of antibody fragments makes them attractive for next-generation precision nanomedicine. Beyond oncology, antibody-conjugated nanocarriers have been developed for inflammatory diseases, cardiovascular disorders, and neurological conditions. For example, antibodies targeting intercellular adhesion molecule 1 (ICAM 1) have been used to direct drug-loaded nanoparticles to inflamed vascular endothelium. Similarly, brain-targeting antibodies such as those recognizing transferrin or insulin receptors facilitate the delivery of nanocarriers across the blood–brain barrier (BBB), which remains one of the most significant obstacles in CNS drug delivery [78]. These examples highlight the versatility of antibody-mediated targeting beyond cancer, enabling nanomedicine to address a wider spectrum of diseases. However, clinical translation of antibody–nanocarrier conjugates is still limited due to potential immunogenicity, high production costs, and challenges in largescale manufacturing. Additionally, the relatively large size of antibodies may hinder deep tissue penetration, limiting their therapeutic impact in solid tumors. Strategies such as using antibody fragments, bispecific antibodies, or engineering synthetic antibody mimetics are being actively explored to address these challenges [79]. Overall, antibody-conjugated nanocarriers provide unmatched specificity and therapeutic potential, but continued innovation is necessary to overcome translational hurdles. Ligand–receptor interactions and antibody-conjugated nanocarriers represent two central pillars of active targeting in smart nanomedicine. While ligands such as folic acid, transferrin, and aptamers provide flexibility and reduced immunogenicity, antibodies offer unmatched specificity for well-characterized biomarkers. Despite biological and translational challenges, including receptor heterogeneity, immunogenicity, and manufacturing complexity, ongoing advances in nanotechnology, bioconjugation techniques, and molecular engineering are steadily addressing these issues. Taken together, these strategies hold strong potential to bring nanomedicine closer to personalized, precise, and clinically translatable therapeutic interventions.

### 3.2. Passive Targeting Mechanisms (EPR Effect)

**Figure 2:**
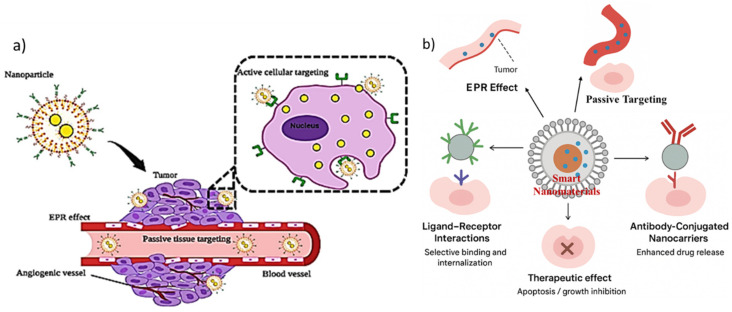
(**a**) Schematic representation of passive and active tumor targeting mechanisms of nanoparticles (adapted from ref. no [87], cc by 4.0). (**b**) Schematic illustration of smart nanomaterials for targeted drug delivery, highlighting both passive targeting via the enhanced permeability and retention (EPR) effect and active targeting strategies through ligand–receptor interactions and antibody-conjugated nanocarriers. These approaches enhance selective binding, cellular internalization, and controlled drug release, ultimately leading to improved therapeutic outcomes such as apoptosis induction and tumor growth inhibition.

Passive targeting through the enhanced permeability and retention (EPR) effect represents one of the most well-documented principles in nanomedicine and cancer therapy. Tumor tissues often display leaky vasculature and impaired lymphatic drainage, which allow nanoscale drug carriers (typically 10–200 nm in size) to preferentially accumulate within the tumor microenvironment (TME) compared to healthy tissues [80]. This selective retention is driven by structural abnormalities in tumor blood vessels, such as irregular basement membranes, fenestrations, and wide inter-endothelial junctions, which permit nanoparticle extravasation. Moreover, impaired lymphatic drainage reduces clearance of macromolecules from the tumor interstitium, thereby prolonging drug exposure at the diseased site [81]. The EPR effect has been central to the development of first-generation nanomedicines, including liposomal doxorubicin (Doxil^®^) and albumin-bound paclitaxel (Abraxane^®^), both of which demonstrated improved pharmacokinetics and reduced systemic toxicity compared to conventional formulations. Nanocarriers exploiting the EPR effect typically improve circulation half-life, maintain higher drug concentrations in tumors, and decrease exposure to healthy tissues. However, heterogeneity of the EPR effect across different tumors and patients remains a critical limitation. Studies have shown that the degree of vascular permeability can vary significantly depending on tumor type, stage, and microenvironmental conditions [82]. Consequently, the clinical predictability of passive targeting remains suboptimal. Several strategies have been proposed to enhance the EPR effect and improve drug delivery efficiency. For instance, nanoparticle size and surface charge have been optimized to achieve longer circulation and better tumor penetration [83]. Polyethylene glycol (PEG) coating of nanocarriers has emerged as a common strategy to avoid opsonization and rapid clearance by the mononuclear phagocyte system (MPS), thereby enhancing blood residence time. Additionally, the use of vasodilators, nitric oxide donors, and angiotensin inhibitors has been explored to transiently increase vascular permeability and thereby improve nanoparticle extravasation into tumors [84]. Despite its advantages, the EPR effect has drawn criticism regarding its clinical translation. Animal tumor models often exhibit exaggerated vascular permeability compared to human tumors, leading to an overestimation of nanoparticle accumulation in preclinical studies [85]. Furthermore, solid tumors with dense extracellular matrix (ECM) and high interstitial fluid pressure (IFP) can impede nanoparticle transport and limit drug distribution within the tumor mass. These barriers underscore the need for combination approaches that integrate passive targeting with active or stimuli-responsive mechanisms. Thus, passive targeting via the EPR effect remains a cornerstone concept in nanomedicine and has provided a foundation for the development of clinically approved formulations. However, variability in tumor vasculature, limited penetration, and inter-patient heterogeneity highlight the need for improved design strategies. Future research should focus on integrating EPR-based delivery with tumor microenvironment modulation and personalized approaches to maximize therapeutic outcomes [86]. Figure 2a, a schematic illustrates how nanoparticles exploit both passive and active mechanisms for tumor targeting. Passive targeting relies on the enhanced permeability and retention (EPR) effect within tumor vasculature, whereas active targeting is achieved through surface functionalization with ligands that specifically bind to tumor-associated receptors, thereby improving therapeutic precision. Figure 2b, highlights the dual strategy of passive and active targeting in nanomaterial-based drug delivery systems, emphasizing their complementary roles in enhancing therapeutic precision. In passive targeting, nanoscale carriers exploit the enhanced permeability and retention (EPR) effect, where leaky tumor vasculature and impaired lymphatic drainage allow preferential accumulation of drugs in diseased tissues. However, to overcome the limitations of heterogeneity in EPR responses, active targeting is introduced by decorating nanocarriers with specific ligands, antibodies, or peptides that recognize overexpressed receptors on cancer cells [87]. This dual mechanism not only improves selective uptake and intracellular trafficking but also minimizes systemic toxicity by sparing healthy tissues. Furthermore, the integration of controlled release mechanisms within these nanocarriers ensures that therapeutic payloads are delivered in a spatiotemporally regulated manner, thereby amplifying apoptotic signaling in malignant cells and suppressing tumor progression. Collectively, the synergy of passive and active targeting exemplifies a rational design principle for next-generation nanomedicines aimed at maximizing efficacy while addressing the challenges of conventional chemotherapy.

### 3.3. Controlled and Stimuli-Triggered Drug Release

One of the defining characteristics of smart nanomaterials is their ability to release therapeutic cargo in a controlled and site-specific manner. Unlike conventional formulations, which often suffer from premature release and systemic toxicity, stimuli-responsive nanocarriers can respond to internal or external triggers to achieve precise drug delivery [18,88]. This feature not only enhances therapeutic efficacy but also reduces off-target effects, improving overall treatment safety. Internal stimuli within the body, such as pH, temperature, redox gradients, and enzymatic activity, have been widely exploited to engineer smart nanocarriers. For example, pH-sensitive liposomes and polymeric micelles release their cargo in the acidic tumor microenvironment (pH 6.5–6.8) or in the even lower pH of endosomes and lysosomes (pH 4.5–5.5) [89,90,91]. Redox-responsive nanomaterials, typically designed with disulfide bonds, undergo cleavage in the high glutathione (GSH) concentrations present in tumor cytoplasm, resulting in rapid drug release [18,92]. Similarly, enzyme-responsive systems utilize overexpressed enzymes in cancer tissues, such as matrix metalloproteinases (MMPs) or cathepsins, to trigger localized release. In addition to internal triggers, external stimuli such as light, magnetic fields, ultrasound, and electric fields provide spatial and temporal control over drug release. Photothermal responsive nanocarriers, often constructed from gold nanoparticles or carbon nanomaterials, can generate heat upon irradiation with near infrared (NIR) light, leading to membrane disruption and drug release [93]. Magnetic field-responsive systems use superparamagnetic iron oxide nanoparticles (SPIONs) to direct carriers to the tumor site and trigger release under alternating magnetic fields. These approaches enable clinicians to control drug activation on demand, which is particularly beneficial for precision oncology [21,94,95,96]. The combination of multiple stimuli-responsive features within a single nanoplatform has gained increasing attention in recent years. For example, hybrid nanocarriers incorporating both pH and redox sensitivity enable sequential release: initial destabilization in acidic extracellular TME followed by cytoplasmic release under reducing conditions [97,98]. Such synergistic designs increase release efficiency and ensure that drugs reach intracellular targets. Controlled drug release systems have demonstrated superior outcomes in preclinical models, including enhanced tumor regression and reduced systemic toxicity. However, clinical translation remains challenging. Many stimuli-responsive systems require complex synthesis and face scalability issues, limiting their industrial applicability. Furthermore, external stimuli such as NIR light and ultrasound have limited penetration depth in human tissues, restricting their applicability to superficial tumors. Nonetheless, continuous progress in nanomaterial design, imaging-guided delivery, and minimally invasive techniques promises to overcome these barriers. Ultimate, controlled, and stimuli-triggered release remains one of the most powerful features of smart nanomaterials in drug delivery [7]. By harnessing both endogenous and exogenous triggers, these systems provide unprecedented precision in therapeutic administration. Future research should emphasize clinically translatable triggers, scalable fabrication methods, and integration with diagnostic modalities to accelerate clinical adoption.

The information presented in Table 3 highlights the diverse range of smart nanomaterials being investigated for targeted drug delivery, emphasizing their design strategies, functional mechanisms, and translational potential. These nanoplatforms, including polymeric nanoparticles, lipid-based carriers, metallic nanostructures, and stimuli-responsive systems, offer unique opportunities to enhance therapeutic efficacy by improving drug solubility, prolonging circulation time, and enabling site-specific release. For instance, pH and enzyme-responsive nanocarriers exploit the abnormal tumor microenvironment to achieve controlled drug release, while ligand-functionalized systems provide precise recognition of overexpressed receptors on diseased cells. Despite these promising advantages, limitations such as potential cytotoxicity, immunogenicity, scalability challenges, and variable in vivo stability remain significant hurdles for clinical translation. Thus, while the examples provided demonstrate considerable progress in tailoring nanomaterials for biomedical applications, further research is needed to optimize their safety, reproducibility, and large-scale manufacturing to bridge the gap between laboratory development and real-world therapeutic implementation.

### 3.4. Case Studies: Recent Advances in Targeted Delivery Systems

Several case studies highlight the transformative potential of smart nanomaterials in targeted drug delivery. These examples underscore the integration of advanced materials, precise targeting, and controlled release strategies to achieve therapeutic success. One notable example is the development of HER2-targeted liposomal doxorubicin for breast cancer therapy. By conjugating trastuzumab (Herceptin^®^) to liposomal surfaces, researchers achieved selective binding to HER2-positive tumor cells, enhancing doxorubicin uptake while minimizing cardiotoxicity [99,100]. Clinical studies demonstrated significantly improved response rates compared to conventional chemotherapy, illustrating the value of antibody-mediated targeting combined with nanoscale delivery. Another example involves polymeric micelles functionalized with folic acid, a ligand that binds to folate receptors overexpressed in many cancers. These micelles encapsulated hydrophobic drugs such as paclitaxel and achieved superior tumor accumulation in murine models. Importantly, the folatetargeted micelles displayed minimal toxicity to healthy tissues, supporting the concept that ligand receptor targeting enhances therapeutic index [101,102]. In addition to cancer therapy, smart nanomaterials have been applied to treat neurological disorders. For instance, transferrin-modified nanoparticles have been engineered to cross the blood–brain barrier (BBB) and deliver therapeutic agents for Alzheimer’s disease and glioblastoma. These systems exploit transferrin receptor-mediated transcytosis to achieve brain penetration, addressing one of the most significant barriers in drug delivery [91,103]. Recent advances also highlight the integration of imaging and therapeutic functionalities in so-called “theranostic” nanoplatforms. For example, iron oxide nanoparticles have been designed to serve both as MRI contrast agents and drug carriers, enabling simultaneous diagnosis and treatment monitoring [104,105]. Similarly, gold nanorods have been employed for combined photothermal therapy and chemotherapy, wherein NIR irradiation not only triggers localized heating but also accelerates drug release at the tumor site. Beyond cancer, targeted delivery systems are being explored for infectious diseases. Nanocarriers functionalized with mannose ligands have been used to target macrophages infected with Mycobacterium tuberculosis, thereby improving antibiotic efficacy and reducing systemic exposure [5,18,106,107]. This approach demonstrates the versatility of smart nanomaterials beyond oncology, extending their relevance to global health challenges. Despite these promising case studies, several challenges persist. Variability in patient responses, large-scale manufacturing difficulties, and regulatory hurdles remain obstacles to widespread clinical translation. Moreover, while preclinical models show significant efficacy, human trials often reveal modest improvements due to differences in disease biology and nanoparticle biodistribution [108]. In conclusion, recent case studies provide compelling evidence that smart nanomaterials can transform targeted drug delivery across oncology, neurology, and infectious diseases. Continued efforts should focus on translational research, patient stratification, and cost-effective production strategies. By bridging laboratory innovation with clinical application, smart nanomaterials hold the potential to redefine precision medicine in the coming decade.

## 4. Smart Nanomaterials in Diagnostic Applications

### 4.1. Nanomaterials for Imaging and Biosensing

The integration of smart nanomaterials into diagnostic platforms has transformed the landscape of modern biomedicine, providing tools with high sensitivity, precision, and multifunctionality. Nanomaterials such as quantum dots, magnetic nanoparticles, gold nanostructures, and carbon-based nanoplatforms have been engineered for imaging and biosensing, allowing real-time monitoring of disease progression and therapeutic response. Their unique physicochemical properties, such as tunable surface chemistry, high surface to volume ratios, and inherent responsiveness to external or biological stimuli, enable both targeted detection and simultaneous therapeutic intervention. Importantly, by combining diagnostic and therapeutic functions, these materials underpin the emerging concept of “theranostics”, offering a bridge between early disease detection and personalized treatment regimens [4,109]. In biosensing applications, nanomaterials enhance sensitivity by providing signal amplification, facilitating recognition of low-abundance biomolecules such as circulating tumor DNA, microRNAs, or disease-specific proteins. For imaging, their optical, magnetic, and electronic properties are tailored to achieve improved spatial resolution, deeper tissue penetration, and lower background interference compared to conventional contrast agents [110,111,112]. Collectively, these advances are establishing smart nanomaterials as indispensable components of precision diagnostics. Thus, the smart nanomaterials thus represent a paradigm shift in diagnostic technologies, not only improving detection limits but also enabling multiplexed analysis, which is critical for complex diseases like cancer or neurodegeneration.

#### 4.1.1. Fluorescence Imaging Nanoprobes

Fluorescence imaging is one of the most powerful tools in biomedical diagnostics due to its high sensitivity, non-invasiveness, and capability for real-time visualization of biomolecular interactions. Smart nanomaterials have substantially expanded the potential of fluorescence imaging by overcoming key limitations such as photobleaching, low quantum yields, and poor tissue penetration. Quantum dots (QDs), carbon dots (CDs), dye-doped silica nanoparticles, and upconversion nanoparticles (UCNPs) represent the most widely studied nanoprobes in this field [95,113,114]. Quantum dots, for instance, provide size-tunable emission wavelengths, high brightness, and superior photostability compared to organic dyes. This allows multiplexed imaging where different QDs can simultaneously detect multiple biomarkers within the same biological environment. Carbon dots, derived from graphitic or polymeric precursors, offer the advantage of low toxicity and facile surface modification, making them particularly suitable for in vivo imaging. Meanwhile, UCNPs, typically based on lanthanide-doped systems, enable excitation in the near infrared (NIR) region, thus achieving deep tissue penetration and minimizing background autofluorescence [6,115]. In addition to their inherent optical properties, smart fluorescence nanoprobes can be engineered to respond to physiological cues such as pH, redox status, or enzyme activity. For example, pH-sensitive carbon dots can selectively illuminate acidic tumor microenvironments, while enzyme-activated fluorophores are turned “on” in the presence of cancer-associated proteases. Such designs provide both diagnostic accuracy and functional insight into the biochemical state of diseased tissue [116]. Recent progress also includes the integration of nanoprobes into multimodal imaging platforms, such as QDs conjugated with MRI agents or UCNPs combined with photothermal sensitizers, enabling simultaneous visualization and therapy. These theranostic platforms are particularly relevant in oncology, where they allow both tumor detection and real-time monitoring of treatment response. Thus, fluorescent nanoprobes, when intelligently engineered, provide a balance of sensitivity, biocompatibility, and multiplexing capability, making them a cornerstone for next-generation diagnostic imaging.

#### 4.1.2. Magnetic Resonance Imaging (MRI) Nanocontrast Agents

Magnetic resonance imaging (MRI) is one of the most widely used clinical imaging modalities, valued for its high spatial resolution and ability to provide detailed anatomical and functional information. However, its sensitivity is relatively low, necessitating the use of contrast agents to enhance signal differences between tissues. Smart nanomaterials have significantly advanced the design of MRI contrast agents by offering superior relaxivity, targeted delivery, and multifunctional diagnostic capabilities [117,118]. Traditional gadolinium-based contrast agents face safety challenges, particularly nephrogenic systemic fibrosis in patients with kidney impairment. In contrast, nanomaterial-based MRI agents such as superparamagnetic iron oxide nanoparticles (SPIONs), manganese oxide nanoparticles, and hybrid core-shell nanostructures exhibit enhanced safety profiles and prolonged circulation times. SPIONs, in particular, are highly effective T2-weighted contrast agents, capable of inducing strong magnetic susceptibility effects, thereby generating high-resolution images of tumors, vascular systems, and inflammatory lesions [119]. Smart nanomaterials also enable active targeting in MRI. By conjugating SPIONs with targeting ligands such as folic acid, antibodies, or peptides, these nanocarriers can selectively accumulate in tumor tissues, increasing diagnostic specificity. Moreover, responsive designs allow contrast agents to alter their magnetic properties in response to physiological triggers such as pH or enzymatic activity. For instance, pH-sensitive SPION assemblies disassemble in acidic tumor microenvironments, leading to enhanced T1 or T2 contrast and improved tumor delineation [120]. Recent innovations include multimodal nanoplatforms that combine MRI with fluorescence, photoacoustic, or computed tomography (CT) imaging. These hybrid systems offer complementary advantages: MRI provides deep tissue anatomical resolution, while fluorescence or photoacoustic imaging delivers high sensitivity and real-time monitoring. For example, manganese-doped UCNPs can act as dual MRI/fluorescence agents, enabling simultaneous tumor detection and intraoperative guidance [121]. Importantly, the biodegradability and clearance of MRI nanocontrast agents are active areas of investigation. Advances in coating strategies, such as using dextran, polyethylene glycol (PEG), or zwitterionic polymers, have improved colloidal stability, reduced immunogenicity, and facilitated renal or hepatic clearance. Such designs are critical to ensure clinical translation and regulatory approval. Concluding remark: Nanomaterial-based MRI contrast agents not only improve imaging sensitivity and specificity but also hold potential as theranostic platforms, bridging molecular diagnostics with personalized therapeutic monitoring.

### 4.2. Theranostic Nanoplatforms: Combined Therapy and Diagnosis

**Figure 3:**
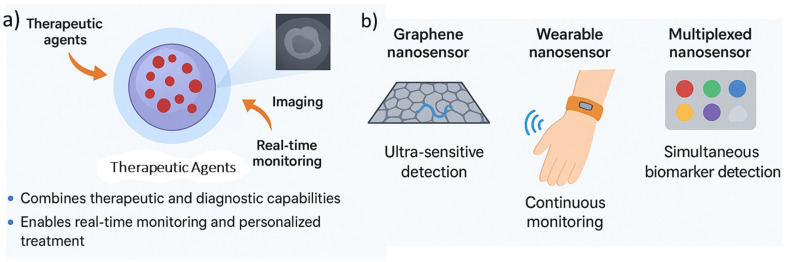
Illustration of theranostic nanoplatforms, (**a**) integrating therapeutic agents with imaging modalities for real-time monitoring, and emerging nanosensor technologies, (**b**) including graphene-based, wearable, and multiplexed systems for ultra-sensitive and continuous disease biomarker detection.

Theranostic nanoplatforms represent a revolutionary frontier in nanomedicine, merging therapeutic and diagnostic capabilities into a single integrated system. Unlike conventional approaches where diagnosis and treatment occur in a sequential manner, theranostics allows real-time disease monitoring while simultaneously administering therapeutic agents. This dual function not only enhances treatment precision but also significantly reduces systemic toxicity by enabling spatiotemporal control over drug delivery and bioimaging [21,122]. At the heart of theranostic systems lies the design of multifunctional nanomaterials engineered to carry therapeutic payloads, respond to external/internal stimuli, and provide imaging contrast for modalities such as MRI, fluorescence, photoacoustic, or positron emission tomography (PET). A key advantage of theranostic nanoplatforms is their ability to facilitate personalized medicine. By offering real-time diagnostic feedback, clinicians can dynamically assess therapeutic efficacy and adjust treatment regimens accordingly. For example, gold-based nanostructures have been extensively explored for theranostics due to their photothermal properties and tunable surface plasmon resonance, enabling simultaneous tumor ablation and optical imaging [123]. Similarly, mesoporous silica nanoparticles (MSNs) functionalized with near infrared (NIR) fluorophores and loaded with chemotherapeutics have demonstrated the capacity to visualize tumor accumulation and trigger drug release upon laser irradiation [124]. These features directly address the limitations of conventional chemotherapy, which often lacks specificity and real-time treatment monitoring. Another promising direction involves the use of magnetic nanoparticles (MNPs). Their inherent magnetic properties make them excellent MRI contrast agents, while surface functionalization allows drug loading and targeted delivery. For instance, doxorubicin-loaded superparamagnetic iron oxide nanoparticles (SPIONs) not only improve tumor visualization under MRI but also enable hyperthermia therapy when exposed to alternating magnetic fields [125]. This multimodality synergistically enhances therapeutic efficacy while minimizing collateral damage to healthy tissues. The incorporation of stimuli-responsive elements has further advanced theranostic platforms. Stimuli such as pH, redox potential, temperature, or enzymatic activity within diseased microenvironments can trigger drug release, while external triggers like ultrasound or light provide additional precision. For instance, pH-sensitive polymer-coated quantum dots have been used for fluorescence imaging and pH-triggered drug release within acidic tumor microenvironments [126]. This ensures that therapeutic activity coincides with diagnostic confirmation of nanoparticle accumulation at the disease site. Figure 3a,b highlights the integration of theranostic nanoplatforms with advanced nanosensing systems, demonstrating their potential in next-generation biomedical applications. The theranostic approach combines therapeutic agents and diagnostic modalities within a single nanosystem, enabling simultaneous disease treatment and real-time monitoring of therapeutic responses. Such platforms often employ multifunctional nanomaterials, such as gold nanoparticles, quantum dots, and polymeric carriers, which allow controlled drug release while providing imaging contrast for modalities like MRI, fluorescence, or photoacoustic imaging. On the other hand, nanosensor technologies depicted in the figure emphasize graphene-based flexible sensors, wearable biosensing devices, and multiplexed detection systems capable of identifying disease biomarkers with ultra-high sensitivity and specificity. Together, these advancements not only accelerate early diagnosis but also pave the way for personalized and precision medicine by enabling continuous health monitoring and tailored therapeutic interventions.

Beyond cancer, theranostic nanoplatforms are increasingly being applied in cardiovascular diseases, neurodegenerative disorders, and infectious diseases. For example, theranostic liposomes carrying anti-inflammatory drugs and ultrasound contrast agents have been developed for real-time monitoring and treatment of atherosclerotic plaques [127]. Similarly, nanostructures integrating anti-amyloid therapeutics with PET tracers are emerging for early detection and treatment of Alzheimer’s disease. These applications highlight the versatility and clinical relevance of theranostics across a wide spectrum of diseases. Despite significant progress, clinical translation remains challenging. Issues such as nanoparticle stability in circulation, immunogenicity, off-target effects, and large-scale reproducibility hinder widespread adoption [128]. Furthermore, regulatory approval pathways for multifunctional nanoplatforms are complex due to the need for simultaneous evaluation of both therapeutic and diagnostic safety. Nevertheless, advances in biocompatible polymers, biodegradable nanocarriers, and surface engineering strategies are progressively overcoming these barriers. Importantly, the integration of artificial intelligence (AI) and machine learning into theranostic design is expected to accelerate optimization by predicting nanoparticle behavior, biodistribution, and treatment outcomes [129,130]. Ultimately, theranostic nanoplatforms exemplify the convergence of therapy and diagnosis, offering a paradigm shift toward precision and personalized medicine. While challenges remain in clinical translation, their ability to monitor, treat, and adapt in real time underscores their transformative potential in modern healthcare. Here, Table 4 summarizes the diverse strategies through which smart nanomaterials are advancing diagnostic and theranostic applications, highlighting their unique capabilities as well as translational challenges. Fluorescence nanoprobes such as quantum dots, carbon dots, and UCNPs enable highly sensitive and multiplexed imaging, while gold nanostructures and graphene-based biosensors provide powerful signal amplification for detecting scarce biomarkers. Similarly, MRI nanocontrast agents like SPIONs and manganese oxide nanoparticles offer high-resolution anatomical imaging with the added benefit of targeted accumulation through ligand conjugation.

Beyond single-function approaches, theranostic nanoplatforms integrate both diagnostic and therapeutic features, enabling real-time treatment monitoring and spatiotemporally controlled drug release, which is particularly valuable in oncology. Stimuli-responsive systems further refine this precision by tailoring activation to disease-specific microenvironments, such as low pH or enzyme-rich niches. Importantly, these platforms are expanding into non-cancer applications, including cardiovascular and neurodegenerative diseases, showcasing their broad potential in precision medicine. However, issues such as in vivo stability, immune clearance, off-target effects, and regulatory complexity remain critical barriers. Overall, the table underscores that while smart nanomaterials offer transformative diagnostic and therapeutic opportunities, addressing safety, scalability, and reproducibility is essential for successful clinical translation.

### 4.3. Emerging Trends in Nanosensors for Disease Monitoring

The continuous monitoring of disease biomarkers in real time is critical for early diagnosis, personalized treatment, and effective management of chronic conditions. Traditional diagnostic techniques, such as ELISA or PCR, though highly accurate, are time-consuming, require specialized infrastructure, and lack the ability to provide real-time feedback. Nanosensors engineered platforms that leverage the unique properties of nanomaterials for ultra-sensitive detection are rapidly emerging as next-generation diagnostic tools for disease monitoring [131]. Their miniaturized scale, high surface-to-volume ratio, and tunable physicochemical properties enable unprecedented sensitivity and specificity, often detecting biomarkers at femtomolar or even attomolar levels. One of the most exciting trends in nanosensors is the use of graphene and other 2D nanomaterials. Graphene’s exceptional electrical conductivity and large surface area allow it to serve as an excellent transducer in electrochemical and optical biosensors. Functionalization with aptamers, antibodies, or peptides enables selective recognition of disease-related biomarkers. For instance, graphene oxide-based nanosensors have been developed for the rapid detection of circulating tumor DNA (ctDNA), offering potential for early cancer diagnosis and continuous monitoring of treatment response [111,132,133,134]. Similarly, transition metal dichalcogenides (TMDs) such as MoS_2_ are being harnessed for their tunable band gaps and photoluminescent properties, advancing fluorescence and field effect transistor (FET) based nanosensors [133,135]. Wearable nanosensors represent another major trend, bridging nanotechnology with digital health. Integrated into fabrics, patches, or implantable devices, wearable nanosensors allow continuous, non-invasive monitoring of physiological parameters such as glucose, lactate, pH, and cortisol. For example, sweat-based nanosensors using ZnO nanowires and carbon nanotubes have demonstrated real-time glucose monitoring for diabetic patients without the need for invasive blood sampling [136,137,138]. Beyond metabolic disorders, wearable nanosensors are being developed for cardiovascular monitoring, detecting biomarkers like troponin I or brain natriuretic peptide (BNP) in sweat or interstitial fluid to predict cardiac events before symptom onset. This convergence of nanotechnology and wearable electronics is significant for preventive medicine and patient-centered care. Another emerging direction is the development of multiplexed nanosensor platforms. Diseases such as cancer and neurodegeneration often involve complex biomarker signatures rather than single indicators. Nanosensors capable of detecting multiple biomarkers simultaneously enable more comprehensive disease profiling. Quantum dot-based nanosensors, for example, utilize distinct emission wavelengths to detect several analytes within a single assay, enhancing diagnostic accuracy and reducing false positives [139]. This multiplexing capability is especially critical in liquid biopsy applications where circulating exosomes, proteins, and nucleic acids must be simultaneously analyzed for effective clinical decision making.

The integration of AI and machine learning is significantly enhancing nanosensor development and application. By analyzing large datasets generated by nanosensor arrays, AI algorithms can identify subtle biomarker patterns and predict disease progression with high accuracy [140]. This is particularly relevant in personalized medicine, where nanosensor-enabled data can guide tailored therapeutic interventions. Furthermore, AI-driven nanosensors are paving the way for automated point-of-care (POC) diagnostics, minimizing the need for expert interpretation and expanding accessibility in low-resource settings. Despite these advances, nanosensor deployment in clinical practice faces hurdles. Challenges include long-term stability of sensor performance, biofouling in complex biological fluids, miniaturization for implantable applications, and ensuring consistent reproducibility in large-scale manufacturing [129,141]. Regulatory and ethical concerns surrounding continuous biomarker monitoring and patient data privacy also require careful consideration. However, innovations such as antifouling surface coatings, biodegradable sensor platforms, and blockchain-based data security systems are emerging as viable solutions [142]. Thus, nanosensors are redefining disease monitoring by enabling highly sensitive, real-time, and non-invasive biomarker detection. Emerging trends such as wearable nanosensors, multiplexed platforms, and AI integration are pushing the boundaries of what is possible in early diagnosis and personalized medicine. Although significant translational challenges remain, the trajectory of nanosensor research indicates a future where continuous health monitoring becomes seamlessly integrated into everyday life, dramatically improving clinical outcomes and patient quality of life.

## 5. Nanomaterials in Regenerative Medicine

### 5.1. Injectable Hydrogels and Nanocomposite Scaffolds

Injectable hydrogels and nanocomposite scaffolds represent one of the most transformative platforms in regenerative medicine. Their tunable physicochemical properties, ease of administration, and capacity to create biomimetic microenvironments make them highly suitable for cell delivery, growth factor encapsulation, and tissue repair. Injectable hydrogels, often derived from natural polymers such as alginate, chitosan, collagen, gelatin, or synthetic polymers like polyethylene glycol (PEG), are designed to transition from liquid to gel state under physiological conditions. This property allows minimally invasive delivery into irregular or hard-to-reach defects, such as bone fractures or neural lesions, where they conform precisely to tissue architecture. The incorporation of nanomaterials, such as graphene oxide, silica nanoparticles, or carbon nanotubes, further enhances their mechanical robustness, bioactivity, and ability to support cellular proliferation and differentiation [102,143]. Conclusively, injectable nanocomposite hydrogels provide a unique combination of injectability, structural reinforcement, and biological functionality that traditional scaffolds cannot achieve. From a biofunctional perspective, nanocomposite hydrogels act as reservoirs for controlled drug and growth factor release. For instance, hydrogel matrices embedded with nanoclays or mesoporous silica nanoparticles can prolong the release kinetics of angiogenic or osteogenic growth factors, ensuring sustained bioactivity at the defect site. This property is especially valuable in musculoskeletal regeneration, where sustained release of bone morphogenetic proteins (BMPs) or vascular endothelial growth factor (VEGF) is critical for bone and vascular repair. Additionally, nanomaterials can enhance electrical conductivity within hydrogels, a feature particularly important in neural and cardiac tissue engineering [144]. For example, hydrogels containing conductive nanofillers such as polypyrrole or carbon nanotubes provide an electroactive environment conducive to neuron regeneration or synchronized cardiac tissue contraction.

**Figure 4:**
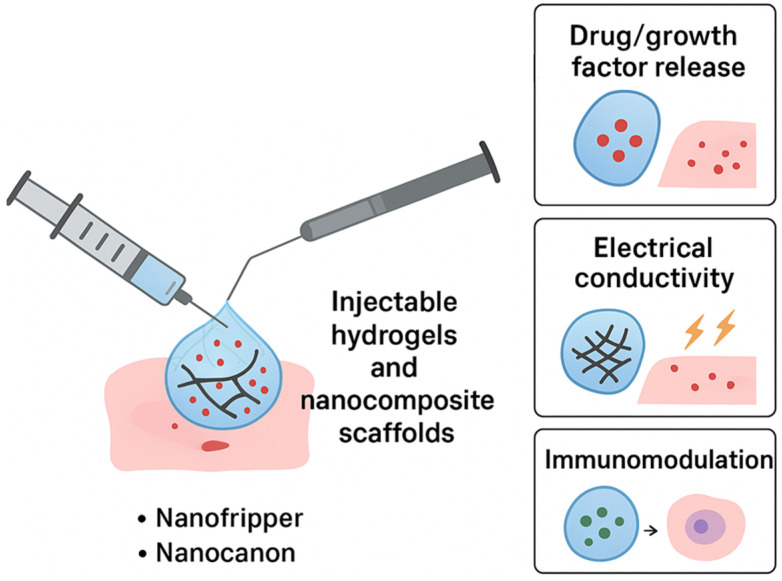
Schematic representation of injectable hydrogels and nanocomposite scaffolds demonstrating their multifunctional roles in drug/growth factor release, electrical conductivity, and immunomodulation.

Thus, the convergence of nanotechnology and hydrogel systems enables fine-tuned spatiotemporal regulation of biological cues essential for functional tissue regeneration. Another critical aspect is the immunomodulatory role of nanocomposite hydrogels. By modifying surface chemistry or embedding bioactive nanoparticles, hydrogels can influence macrophage polarization toward pro-healing M2 phenotypes. For example, cerium oxide nanoparticles within hydrogels provide antioxidant activity, reducing reactive oxygen species (ROS) levels and mitigating chronic inflammation at injury sites. This immune modulatory effect fosters an environment favorable for tissue integration and long-term functional recovery [5,145,146]. Thus, the injectable hydrogels and nanocomposite scaffolds represent a paradigm shift in regenerative medicine by simultaneously addressing structural, biochemical, and immunological requirements of tissue repair. Figure 4 highlights the multifunctional applications of injectable hydrogels and nanocomposite scaffolds in regenerative medicine and therapeutic delivery. These advanced biomaterials serve as versatile platforms capable of controlled drug and growth factor release, enabling localized and sustained therapeutic effects at the target site. The incorporation of conductive nanomaterials further imparts electrical responsiveness, which is particularly beneficial for tissue engineering in electrically active tissues such as cardiac and neural systems. Additionally, their ability to modulate immune responses through embedded bioactive cues makes them promising candidates for promoting tissue repair and integration while minimizing adverse inflammatory reactions. Together, these properties underscore the potential of nanocomposite hydrogels, such as Nanofripper and Nanocanon systems, to bridge the gap between conventional scaffolds and next-generation biofunctional implants. Nanofripper: A molecular component, or artificial molecular machine (AMM), with a design that allows it to grip, pull, or unzip other molecules. Its function could involve manipulating complex molecules, such as protein folding or DNA processing, on the scaffold. Nanocanon: A molecular component designed to rapidly propel or deliver other molecules. It could be used for targeted delivery of therapeutic molecules, such as drugs or genes, into specific cells or tissues attached to the scaffold.

### 5.2. Nanoparticles for Stem Cell Therapy

Nanoparticles (NPs) are increasingly being applied in stem cell therapy as powerful tools to enhance therapeutic efficacy, regulate stem cell fate, and monitor transplanted cells in vivo. The integration of nanotechnology with stem cell biology allows precise manipulation of cellular microenvironments and intracellular processes. One significant application is the use of NPs as vehicles for gene and drug delivery to stem cells. For example, gold nanoparticles, mesoporous silica nanoparticles, and polymeric NPs can deliver genetic material or bioactive molecules to stem cells, promoting differentiation into specific lineages such as osteoblasts, neurons, or cardiomyocytes [95,147]. These nanoparticle-based delivery systems provide high loading capacity, controlled release, and efficient cellular uptake, which ensures reliable modulation of stem cell behavior. Conclusively, NPs empower stem cell therapy by bridging biological limitations with targeted nano-enabled interventions. Magnetic nanoparticles (MNPs) offer unique advantages in guiding stem cell migration and localization. By applying an external magnetic field, MNP-labeled stem cells can be directed toward injured tissues, improving homing efficiency and therapeutic outcomes. For example, MNPs have been employed to direct mesenchymal stem cells (MSCs) to ischemic myocardial tissues, significantly enhancing cardiac repair and vascularization. Similarly, superparamagnetic iron oxide nanoparticles (SPIONs) are widely studied for their role in tracking stem cells through magnetic resonance imaging (MRI), enabling non-invasive monitoring of cell survival, distribution, and integration after transplantation [148]. This dual role of MNPs in both functional enhancement and diagnostic tracking underscores their potential as indispensable components of regenerative medicine. Beyond delivery and tracking, NPs also serve as bioactive modulators that directly influence stem cell differentiation. For example, nanoscale hydroxyapatite and bioactive glass nanoparticles mimic the mineralized extracellular matrix of bone, promoting osteogenic differentiation of MSCs. Carbon-based nanomaterials, such as graphene and carbon nanotubes, provide nanoscale topographical and electrical cues that favor neuronal differentiation. Furthermore, NPs with antioxidant or anti-inflammatory properties, such as cerium oxide NPs, protect transplanted stem cells from hostile microenvironments, increasing their survival rate post-implantation [6,19,149,150]. Ultimately, NPs enable a multifaceted enhancement of stem cell therapy by serving as delivery vectors, imaging agents, and bioactive regulators, thus overcoming key barriers in stem cell-based regenerative medicine.

### 5.3. Applications in Musculoskeletal and Neural Regeneration

The musculoskeletal and nervous systems are among the most challenging tissues to regenerate due to their structural complexity, poor intrinsic healing capacity, and high susceptibility to chronic inflammation. Nanomaterials have emerged as critical enablers of regenerative strategies in these systems. In musculoskeletal regeneration, nanostructured scaffolds mimic the hierarchical architecture of bone and cartilage. For instance, nanohydroxyapatite incorporated into polymeric scaffolds provides both mechanical reinforcement and osteoinductive signaling, closely resembling natural bone mineral composition. These scaffolds also allow for controlled release of growth factors, such as BMP 2, thereby accelerating bone healing in critical-size defects [102,151]. Additionally, carbon-based nanomaterials and metallic nanoparticles have been integrated into scaffolds to enhance mechanical resilience and antimicrobial activity, reducing implant-associated infections. Thus, nanomaterials provide structural, biochemical, and antimicrobial functions in musculoskeletal tissue engineering. Cartilage regeneration presents unique challenges due to its avascular nature, making spontaneous repair extremely limited. Nanocomposite hydrogels with embedded nanocellulose or graphene oxide have shown promise in promoting chondrocyte proliferation and extracellular matrix deposition. Similarly, electrospun nanofibers mimic the fibrous architecture of native cartilage and serve as platforms for delivering chondrogenic growth factors. These nanoscale interventions ensure the formation of functional cartilage with improved biomechanical integrity. Importantly, integrating nanomaterials within cartilage scaffolds helps resist mechanical wear and tear, enhancing long-term durability of engineered tissue [152]. Conclusively, nanotechnology-driven strategies provide new opportunities to address the limitations of musculoskeletal repair. In neural regeneration, nanomaterials offer unique advantages owing to their ability to mimic the nanoscale architecture of neuronal extracellular matrix and provide electrical cues necessary for neuron communication. Conductive nanomaterials, such as carbon nanotubes, graphene, and conductive polymers, have been widely used to stimulate axonal outgrowth and synapse formation. For example, aligned nanofiber scaffolds integrated with conductive nanomaterials provide directional guidance for axon regeneration while promoting electrophysiological activity. Moreover, nanoparticles serve as carriers for neurotrophic factors, ensuring sustained release and localized delivery to damaged neural tissues [111,153]. This controlled biochemical signaling significantly enhances neuronal survival and regeneration. Therefore, nanotechnology offers not only physical scaffolding but also bioelectrical and biochemical modulation for successful neural repair.

### 5.4. Biomimetic Nanomaterials for Tissue Engineering

Biomimetic nanomaterials are designed to replicate the structural, chemical, and biological characteristics of native extracellular matrices (ECM), providing a supportive environment for tissue development and integration. One of the key principles of biomimicry in nanomaterials is the recreation of nanoscale topographies and biochemical cues that regulate cell adhesion, proliferation, and differentiation. For instance, self-assembling peptide nanofibers form ECM-like structures that closely resemble collagen fibrils, guiding cellular organization and tissue regeneration [154]. Similarly, nanostructured calcium phosphate coatings mimic bone mineral, promoting osteointegration of orthopedic implants. By imitating the functional aspects of natural ECM, biomimetic nanomaterials achieve superior cell–material interactions and accelerate tissue regeneration. Another critical advancement in biomimetic nanomaterials is their ability to incorporate bioactive signaling molecules. For example, nanocarriers embedded within biomimetic scaffolds can deliver growth factors, nucleic acids, or small molecules in a controlled manner, simulating natural paracrine signaling within tissues. Moreover, dynamic biomimetic systems have been developed to respond to physiological stimuli, such as pH or mechanical stress, thereby providing adaptive support to regenerating tissues. This adaptability is especially relevant in dynamic environments like musculoskeletal and cardiovascular tissues, where mechanical loading is integral to function [155]. Conclusively, the integration of responsive functionalities within biomimetic nanomaterials bridges the gap between static scaffolds and living tissues. Importantly, biomimetic nanomaterials also address immune compatibility and long-term integration challenges. By modifying surface chemistry with biomolecules such as RGD peptides or heparin mimicking moieties, these materials promote selective cell adhesion while minimizing foreign body reactions. Recent studies have shown that biomimetic nanofiber scaffolds can modulate macrophage phenotypes, steering immune responses toward tissue remodeling rather than fibrosis. This immunomodulatory property enhances long-term graft survival and functionality [156]. In conclusion, biomimetic nanomaterials provide a next-generation strategy in regenerative medicine, combining structural mimicry, biochemical signaling, and immune compatibility to achieve clinically translatable outcomes.

Table 5 provides a comprehensive overview of how nanomaterials are increasingly integrated into regenerative medicine, emphasizing their multifunctional roles in promoting tissue repair and functional recovery. Various strategies, including scaffold-based delivery, growth factor immobilization, and stem cell modulation, demonstrate the versatility of nanomaterials in guiding cellular behavior and enhancing tissue regeneration. Their nanoscale features allow precise interaction with biological systems, thereby improving biocompatibility, mechanical strength, and controlled release of therapeutic agents. At the same time, these materials offer distinct advantages such as mimicking the extracellular matrix, enhancing angiogenesis, and accelerating healing processes across bone, neural, and cardiac tissues. However, despite these promising applications, limitations such as potential cytotoxicity, immune responses, scalability of fabrication, and long-term safety remain major challenges. Thus, while nanomaterials hold great potential to revolutionize regenerative medicine, further optimization and clinical validation are critical to ensure safe and effective translation from laboratory to patient care.

## 6. Clinical Translation and Challenges

### 6.1. Safety, Toxicity, and Immunogenicity Issues

The clinical translation of nanomaterials for therapeutic applications has been largely limited by concerns regarding safety, toxicity, and immunogenicity. Despite the promising efficacy of nanomedicine demonstrated in preclinical studies, several uncertainties remain regarding how nanomaterials interact with complex human physiological systems. The unique physicochemical properties of nanomaterials, such as their size, shape, surface chemistry, and charge, can impart both therapeutic benefits and toxicological risks, making a clear evaluation essential for clinical use [11,157]. A primary challenge lies in the biodistribution and clearance of nanomaterials. Nanoparticles smaller than 10 nm are rapidly eliminated through renal filtration, while those larger than 200 nm tend to accumulate in the liver and spleen due to reticuloendothelial system (RES) uptake. While such organ accumulation can enhance local therapy in specific cases, it often leads to concerns of off-target toxicity and long-term retention. For example, gold nanoparticles (AuNPs) and carbon-based nanomaterials have been reported to persist in organs for extended durations, raising potential risks of chronic toxicity [158]. Another critical issue is oxidative stress and inflammation triggered by reactive oxygen species (ROS) generated upon nanoparticle cell interactions. Transition metal oxides such as ZnO or TiO_2_ can cause mitochondrial damage and DNA fragmentation through ROS overproduction [159]. Similarly, cationic surface charges, while beneficial for enhancing cellular uptake, often disrupt cell membranes and increase cytotoxicity. Consequently, surface modification strategies such as PEGylation, zwitterionic coatings, or biomimetic cloaking are widely used to mitigate toxicity and reduce immune recognition [160]. Immunogenicity is a significant barrier in clinical translation. Nanomaterials may activate the complement system, leading to complement activation-related pseudoallergy (CARPA). This hypersensitivity reaction has been reported for liposomal formulations such as Doxil^®^, requiring careful preclinical immunological testing [161]. Additionally, nanoparticle-induced cytokine storms pose serious risks in systemic administration, particularly in immunocompromised patients. Personalized immunotoxicity assessments are therefore critical to predict patient-specific risks [162]. Long-term toxicity remains poorly understood due to the lack of standardized chronic exposure studies. Most toxicity assessments focus on short-term cytotoxicity in vitro or acute animal models, which fail to capture long-term effects such as carcinogenicity, reproductive toxicity, or transgenerational risks [163]. Emerging evidence suggests that nanomaterials may interfere with genetic regulation, epigenetic modifications, and stem cell differentiation, which could have implications for developmental biology and fertility [164]. Thus, addressing safety and immunogenicity concerns requires integrated approaches: (1) developing standardized testing protocols, (2) designing biodegradable and stimuli-responsive nanomaterials, and (3) utilizing advanced in vitro organ-on-chip and in silico models for predictive toxicology. In conclusion, while nanomaterials hold strong therapeutic promise, systematic toxicity evaluations and immunological profiling remain indispensable for ensuring safe and effective clinical translation.

### 6.2. Manufacturing and Scalability Challenges

**Figure 5:**
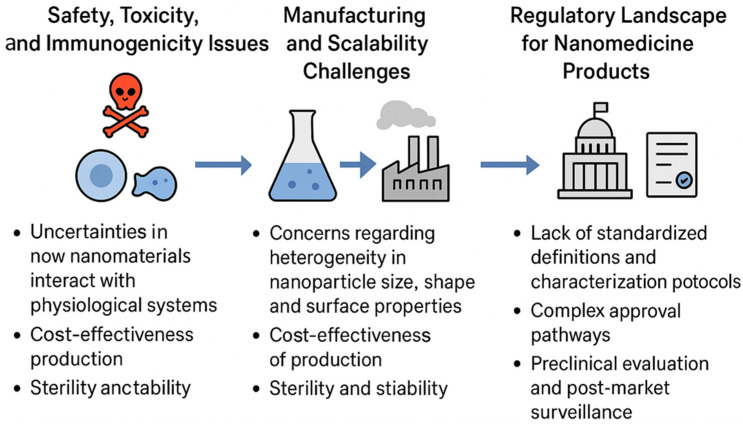
Infographic illustrating the clinical translation pathway of injectable biopolymer-based hydrogels, highlighting their design, fabrication, preclinical evaluation, regulatory approval, and therapeutic applications in regenerative medicine.

The translation of nanomaterials from laboratory synthesis to clinical scale production faces major obstacles in manufacturing consistency, cost efficiency, and scalability. While laboratory-scale syntheses often rely on highly controlled conditions, the upscaling of nanomaterials without compromising their structural integrity, functionality, and batch-to-batch reproducibility remains a pressing challenge [165]. One of the primary concerns is the heterogeneity in nanoparticle size, shape, and surface properties during scale-up. For example, liposomes, polymeric nanoparticles, and metallic nanostructures often require precise control over physicochemical features to achieve reproducible pharmacokinetics and biodistribution. Minor variations in size distribution or surface charge can significantly alter therapeutic outcomes. Current scale-up techniques, such as microfluidic-based synthesis and high-pressure homogenization, are being increasingly explored to achieve better reproducibility [166]. Another challenge is cost-effectiveness and process optimization. The synthesis of high-quality nanomaterials often involves expensive reagents, energy-intensive conditions, and labor-intensive purification steps. For instance, large-scale production of gold nanoparticles or graphene oxide is hindered by high material and processing costs. Similarly, nanocarriers requiring complex functionalization, such as ligand-targeted or stimuli-responsive designs, introduce additional manufacturing burdens [167]. Sterility, stability, and storage are equally crucial considerations. Many nanocarriers, especially lipid and polymer-based systems, are prone to aggregation, hydrolysis, or oxidation, leading to reduced efficacy and shelf life. Lyophilization with cryoprotectants, optimized storage conditions, and encapsulation strategies have been employed to enhance long-term stability, though these add further manufacturing complexity [168]. Good manufacturing practices (GMP) compliance presents another regulatory hurdle. Ensuring sterility, reproducibility, and traceability of each batch requires sophisticated facilities and quality control measures. Analytical challenges include characterizing nanomaterials’ surface chemistry, coating thickness, and in vivo degradation pathways at scale. Emerging characterization tools such as single particle tracking, nanoparticle flow cytometry, and synchrotron-based imaging are gradually being integrated into GMP pipelines [169]. The future of scalable nanomedicine production likely depends on automated, high-throughput platforms that minimize human error and variability. Microfluidics-based continuous manufacturing and AI-driven process optimization are being proposed as solutions to streamline synthesis, improve reproducibility, and reduce costs [170]. In conclusion, while nanomaterials show strong therapeutic potential, scalable and standardized production remains a critical bottleneck to their clinical translation, requiring collaborative efforts from materials scientists, engineers, and regulatory agencies. Figure 5 presents a schematic overview of the clinical translation pathway for injectable biopolymer-based hydrogels, emphasizing the interconnected stages from fundamental material design to therapeutic deployment. The process begins with the rational selection and engineering of natural polymers, where biocompatibility, injectability, and tunable physicochemical properties are optimized to ensure clinical viability. Subsequent fabrication strategies, including crosslinking approaches and incorporation of bioactive cues, are critical in tailoring hydrogels for specific therapeutic targets. Preclinical evaluation in vitro and in vivo plays a central role in validating their safety, biodegradability, and functional efficacy, while scaling up production requires adherence to good manufacturing practices (GMP) to ensure reproducibility. The regulatory framework serves as a pivotal checkpoint, demanding comprehensive safety and performance data before approval for human use. Finally, successful clinical integration of these hydrogels enables their application in regenerative medicine, drug delivery, and minimally invasive therapies, bridging the gap between laboratory innovation and real-world healthcare solutions.

### 6.3. Regulatory Landscape for Nanomedicine Products

The regulatory framework for nanomedicines remains under continuous evolution as agencies such as the FDA (U.S.), EMA (Europe), and PMDA (Japan) attempt to address the unique challenges posed by nanotechnology-based therapeutics. Unlike conventional small molecules or biologics, nanomedicines often exhibit complex pharmacokinetics, dynamic interactions with biological systems, and multifunctional mechanisms of action, which complicate regulatory assessments [171]. One of the fundamental issues is the lack of standardized definitions and characterization protocols for nanomaterials. For instance, the FDA defines nanotechnology based on dimensions within 1–100 nm, but many clinically relevant nanocarriers, such as liposomes and polymeric micelles, exceed this size range yet retain nanoscale properties [172]. This lack of clarity complicates regulatory classification, leading to inconsistent approval pathways across regions. Preclinical evaluation presents another challenge. Regulators require extensive safety, toxicity, and immunogenicity data, yet conventional assays may not accurately predict nanomaterials’ in vivo behavior. To address this, agencies recommend a case-by-case approach, focusing on physicochemical characterization, biodistribution, and long-term safety [173]. However, the absence of harmonized international guidelines often slows down approval timelines. The regulatory pathway also depends on whether the nanomedicine is classified as a drug, biologic, device, or combination product. For example, liposomal doxorubicin (Doxil^®^) was approved under conventional drug frameworks, whereas nanoparticle-coated stents are classified as combination products, requiring additional device-specific evaluations. This complexity adds uncertainty for developers navigating approval processes [174]. To address these challenges, regulatory agencies are increasingly adopting adaptive and risk-based frameworks. The FDA’s Nanotechnology Task Force, EMA’s Reflection Paper on Nanomedicines, and WHO initiatives aim to establish international harmonization and encourage early dialogue between developers and regulators. Furthermore, the integration of real-world evidence, post-marketing surveillance, and digital health tools is being explored to monitor nanomedicines’ long-term performance [175]. Ultimately, successful translation requires collaborative engagement among researchers, industry stakeholders, and regulators to establish clear guidelines for nanomedicine characterization, clinical testing, and lifecycle monitoring. In conclusion, while regulatory uncertainty remains a hurdle, progressive harmonization of global regulatory frameworks offers a path toward safer and faster clinical translation of nanomaterials.

### 6.4. Case Studies of Clinically Approved Nanomaterial-Based Therapeutics

Despite significant challenges, several nanomaterial-based therapeutics have achieved clinical approval, demonstrating the translational potential of nanomedicine. These case studies highlight both the successes and the challenges associated with bringing nanotechnology into mainstream healthcare. One of the earliest and most successful nanomedicines is liposomal doxorubicin (Doxil^®^), approved by the FDA in 1995. By encapsulating doxorubicin in a PEGylated liposomal carrier, Doxil^®^ enhances drug accumulation in tumors via the enhanced permeability and retention (EPR) effect while reducing cardiotoxicity—a major side effect of conventional doxorubicin [176]. The clinical success of Doxil^®^ paved the way for other liposomal formulations such as DaunoXome^®^ and Onivyde^®^. Albumin-bound paclitaxel (Abraxane^®^) represents another landmark approval. By conjugating paclitaxel to albumin nanoparticles, Abraxane^®^ bypasses the need for toxic solvents like Cremophor EL, improves solubility, and enhances tumor penetration. This formulation has shown significant clinical benefits in breast, lung, and pancreatic cancers, demonstrating how nanocarriers can overcome long-standing drug delivery barriers [177]. Metal-based nanomedicines have also reached clinical application. Feraheme^®^ (ferumoxytol), an iron oxide nanoparticle, was approved as an intravenous iron replacement therapy and later investigated as an MRI contrast agent. Its dual therapeutic and diagnostic role underscores the potential of nanomaterials in theranostics [178]. Similarly, nanostructured lipid carriers have been employed in mRNA-based COVID-19 vaccines (Pfizer BioNTech and Moderna), representing the most impactful real-world success of nanotechnology in recent years [179]. However, not all clinical experiences have been favorable. Some nanoparticle formulations failed due to unpredictable pharmacokinetics, toxicity, or lack of a significant efficacy advantage over conventional drugs. For example, certain polymeric nanoparticle formulations of camptothecin derivatives were discontinued in late-stage trials due to safety concerns [180]. These failures highlight the need for better predictive preclinical models and more stringent safety evaluations. Collectively, these case studies reveal that while nanomedicines have achieved remarkable milestones, clinical translation remains selective and highly context-dependent. The key factors determining success include clear therapeutic advantages, well-characterized safety profiles, and scalable manufacturing processes. In conclusion, the approved nanomedicine products provide not only validated therapeutic options but also roadmaps and lessons for the next generation of nanomaterials in clinical translation. Table 6 provides a comparative overview of the clinical translation of nanomaterials, highlighting the multifaceted barriers that impede their successful transition from bench to bedside. The table delineates unresolved challenges such as toxicity, scalability, reproducibility, and long-term biocompatibility, which remain critical concerns despite significant preclinical advances. It further summarizes innovative strategies, including surface functionalization, hybrid nanostructures, and precision targeting, which are being developed to mitigate these obstacles and enhance clinical outcomes.

In addition, evolving regulatory paradigms are addressed, reflecting the increasing need for harmonized international guidelines and rigorous evaluation frameworks tailored specifically to nanoscale therapeutics. Finally, instructive case studies are presented to illustrate both successful and stalled translational efforts, thereby offering valuable insights into the practical realities of nanomedicine development and underscoring the interplay between scientific innovation, regulatory oversight, and clinical feasibility.

## 7. Future Perspectives and Opportunities

### 7.1. Personalized Nanomedicine

The field of personalized nanomedicine represents a measurable improvement approach in healthcare, aiming to tailor therapeutic interventions to individual patient profiles. Unlike traditional “one size fits all” therapies, personalized nanomedicine integrates genomic, proteomic, and metabolomic data with nanotechnology-enabled delivery systems, allowing for highly targeted and patient-specific treatments [181]. Smart nanomaterials, with tunable size, surface chemistry, and stimuli-responsive properties, are particularly suited for this paradigm because they can be engineered to selectively interact with diseased tissues while sparing healthy cells. A key advantage of personalized nanomedicine lies in its ability to overcome interpatient variability in drug response. Genetic polymorphisms, differences in tumor microenvironments, and individual metabolic profiles can significantly influence therapeutic efficacy and toxicity. For instance, nanoparticles functionalized with patient-specific ligands, such as antibodies targeting overexpressed receptors, allow selective delivery of chemotherapeutics, minimizing off-target effects [182]. Similarly, nanocarriers can be tailored to release drugs in response to endogenous stimuli such as pH, redox potential, or enzymatic activity, reflecting the unique pathological environment of each patient. These features collectively enhance treatment precision and reduce systemic toxicity. Beyond targeted therapy, personalized nanomedicine enables dynamic monitoring of treatment outcomes. Theranostic nanoplatforms that combine drug delivery and diagnostic imaging allow clinicians to assess therapeutic efficacy in real time and adjust dosing regimens accordingly. For example, MRI visible polymeric nanoparticles loaded with chemotherapeutics can simultaneously visualize tumor regression and provide feedback on drug distribution, enabling a feedback-driven approach to therapy optimization [183]. This integration of therapy and monitoring is particularly valuable in oncology, where tumor heterogeneity often leads to differential drug responses. Challenges in personalized nanomedicine include the need for rapid and accurate patient profiling, scalable production of individualized nanocarriers, and robust preclinical evaluation. High-throughput screening methods, microfluidic-based nanoparticle fabrication, and computational modeling are emerging as tools to address these challenges [184]. Importantly, ethical and privacy considerations surrounding the collection and use of patient-specific omics data must be carefully managed to ensure responsible implementation. Thus, personalized nanomedicine represents a paradigm shift toward precision healthcare. By leveraging the unique capabilities of smart nanomaterials, it promises enhanced efficacy, reduced toxicity, and real-time adaptability in clinical interventions, marking a significant step toward truly individualized therapy.

### 7.2. Artificial Intelligence and Smart Nanomaterials

The integration of artificial intelligence (AI) with smart nanomaterials offers unprecedented opportunities for the design, optimization, and application of nanomedicine. AI algorithms, particularly machine learning and deep learning approaches, can analyze large datasets generated during nanoparticle synthesis, characterization, and in vivo testing, enabling predictive modeling of nanoparticle behavior and therapeutic outcomes [185]. One major application of AI is in nanomaterial design. Traditional trial and error methods are often time-consuming and resource-intensive. AI-driven predictive models can optimize nanoparticle size, shape, surface chemistry, and functionalization strategies to achieve desired pharmacokinetics, biodistribution, and targeting efficacy. For example, supervised learning algorithms have been used to correlate nanoparticle surface properties with cellular uptake efficiency, allowing the rational design of targeted nanocarriers [186]. This approach accelerates the development of personalized nanomedicine by enabling patient-specific nanocarrier optimization. AI is also instrumental in smart drug delivery. Reinforcement learning algorithms can be employed to predict the optimal release profiles of stimuli-responsive nanocarriers, accounting for individual patient physiology and disease microenvironment. Similarly, AI-enabled image analysis allows real-time monitoring of theranostic nanoparticles, detecting subtle changes in tumor size or drug accumulation that may not be apparent through conventional imaging techniques [187]. Beyond drug delivery, AI enhances predictive toxicology and safety assessment. By integrating high-dimensional datasets from in vitro assays, in vivo models, and clinical observations, machine learning models can predict immunogenicity, cytotoxicity, and long-term nanoparticle behavior. This predictive capability reduces the need for extensive animal testing and enables safer clinical translation of nanomedicines [188]. However, challenges exist in integrating AI with nanomedicine, including data heterogeneity, a lack of standardized reporting, and the interpretability of complex models. Efforts to address these challenges include the development of large, curated databases of nanomaterial properties, standardized experimental protocols, and interpretable AI algorithms [189]. In conclusion, the convergence of AI and smart nanomaterials provides data-driven strategies for the rational design, optimization, and monitoring of nanomedicine, enabling more effective, safe, and personalized therapeutic interventions. This integration is poised to redefine the landscape of nanomedicine research and clinical application.

### 7.3. Green and Sustainable Synthesis of Smart Nanomaterials

**Figure 6:**
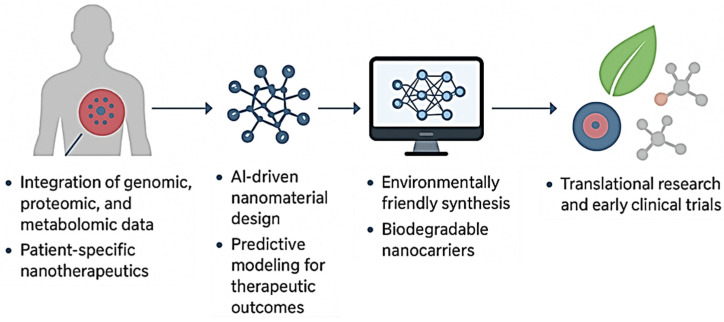
Schematic representation of future perspectives in smart nanomedicine, emphasizing the shift toward personalized treatments through the integration of genomic, proteomic, and metabolomic data. Advances include AI-assisted design of nanomaterials, eco-friendly synthesis using biodegradable carriers, and stronger efforts to translate laboratory innovations into clinical applications.

Sustainability in nanomaterial synthesis has emerged as a critical concern due to environmental and health considerations associated with conventional chemical and physical fabrication methods. Green and sustainable strategies aim to reduce toxic reagents, energy consumption, and hazardous byproducts while maintaining or enhancing nanoparticle functionality [190]. Biogenic synthesis approaches utilize plant extracts, microorganisms, and biomolecules as reducing and stabilizing agents. For example, gold and silver nanoparticles have been synthesized using plant phytochemicals, which act both as reducing agents and surface capping molecules, eliminating the need for hazardous chemicals [191]. These methods not only reduce environmental impact but also confer enhanced biocompatibility due to natural surface functionalization. Another approach involves solvent-free or low-energy fabrication techniques, such as mechanochemical synthesis, microwave-assisted synthesis, and supercritical fluid processing. These methods reduce energy requirements and minimize solvent-related waste. For instance, microwave-assisted synthesis of metal oxide nanoparticles achieves rapid nucleation and uniform size distribution while significantly reducing reaction times and energy consumption [192]. In addition to synthesis, the biodegradability and recyclability of nanomaterials are important for sustainability. Biodegradable polymers such as poly(lactic co-glycolic acid) (PLGA), chitosan, and gelatin have been widely employed to create smart nanocarriers that degrade into non-toxic byproducts, reducing long-term environmental accumulation [193]. Similarly, recycling and recovery strategies for metal-based nanomaterials are being developed to minimize environmental release and promote circular economy principles. Green synthesis also aligns with regulatory and societal expectations, as environmentally benign nanomedicines are more likely to gain acceptance and support for large-scale production. Despite these advantages, challenges include scalability, batch reproducibility, and fine-tuning physicochemical properties to achieve therapeutic efficacy [194]. Thus, the green and sustainable synthesis represents a responsible and forward-looking approach to developing smart nanomaterials, balancing therapeutic performance with environmental stewardship. Adoption of these strategies is expected to facilitate safer, cost-effective, and environmentally compatible nanomedicine development [195]. Figure 6 highlights the emerging directions in smart nanomedicine, where the convergence of advanced technologies and biological insights is expected to transform therapeutic strategies. A key focus is the move toward personalized treatments, achieved by integrating patient-specific genomic, proteomic, and metabolomic information to design tailored nanocarriers with improved precision and efficacy. The incorporation of artificial intelligence (AI) enables data-driven optimization of nanomaterial properties, facilitating faster and more accurate development of multifunctional platforms. At the same time, eco-friendly and sustainable synthesis approaches, such as the use of biodegradable carriers, address environmental and safety concerns while ensuring biocompatibility. Importantly, significant attention is being directed toward bridging the gap between laboratory research and clinical translation, with efforts to overcome regulatory, scalability, and reproducibility challenges. Together, these advancements outline a roadmap for the next generation of nanomedicine, aiming for safer, smarter, and patient-specific healthcare solutions.

### 7.4. Bridging the Gap Between Research and Clinical Application

Despite significant advances in nanomedicine, a persistent gap exists between laboratory research and clinical application, often termed the “valley of death” in translational research. Factors contributing to this gap include challenges in reproducibility, regulatory uncertainty, scalability, and the complex biological behavior of nanomaterials in humans [196]. Bridging this gap requires multidisciplinary collaboration among chemists, materials scientists, biologists, clinicians, and regulatory experts. Early integration of clinical considerations into nanoparticle design can improve the likelihood of successful translation. For example, tailoring nanocarriers to match human physiological conditions, understanding patient-specific variability, and incorporating feedback from clinical trials into iterative design cycles can enhance clinical readiness [197]. Standardization and reproducibility are critical. Establishing robust protocols for synthesis, characterization, and preclinical evaluation ensures that findings are consistent across laboratories and can be reliably scaled up for clinical production. Advanced characterization tools, such as single particle tracking and organ-on-chip models, provide more predictive insights into nanoparticle behavior in vivo [198]. Furthermore, early engagement with regulatory agencies can streamline the translation process. Regulatory guidance on nanomedicines is evolving, but proactive interaction helps identify safety, efficacy, and manufacturing requirements early in development, reducing delays in approval [199]. Finally, integrating patient-centric design and digital health tools enhances clinical applicability. Nanomedicine platforms that incorporate monitoring, diagnostics, and adaptive dosing strategies can better align with real-world therapeutic needs, increasing adoption in clinical practice [200]. Thus, bridging the gap between research and clinical application requires holistic strategies combining multidisciplinary design, standardization, regulatory foresight, and patient-centered approaches, ultimately ensuring that innovations in smart nanomaterials translate into meaningful clinical impact.

### 7.5. Clinical Translation and Regulatory Perspectives

Despite the rapid progress in nanomaterial design and biomedical applications, the clinical translation of smart nanomaterials remains limited. One of the main challenges lies in large-scale manufacturing, where maintaining consistent particle size, surface chemistry, and functional performance is difficult when moving from laboratory synthesis to industrial production. Reproducibility and batch-to-batch uniformity are essential to ensure predictable pharmacokinetics and therapeutic outcomes. Another major barrier is the comprehensive assessment of long-term toxicity and biodistribution, particularly for non-biodegradable inorganic nanoparticles that may accumulate in organs such as the liver, spleen, and lungs. Pharmacokinetic and pharmacodynamic profiling are critical for understanding how nanomaterials circulate, distribute, and are cleared from the body, yet these parameters often vary depending on surface chemistry and biological milieu [201]. Immunogenicity is another growing concern, as repeated dosing of certain nanocarriers (e.g., PEGylated or protein-coated systems) can induce immune recognition or accelerated blood clearance. On the regulatory front, most existing frameworks were developed for conventional pharmaceuticals and do not adequately address the complexity of multifunctional nanomedicines. Agencies such as the FDA and EMA now emphasize detailed physicochemical characterization, standardized toxicity testing, and lifecycle management of nanoproducts. However, the absence of harmonized global guidelines continues to delay approval timelines. Overcoming these barriers will require close collaboration between material scientists, clinicians, and regulatory authorities to develop standardized manufacturing practices and validated evaluation methods. Such coordinated efforts are essential to bridge the gap between experimental innovation and safe, effective clinical translation.

**Figure 7:**
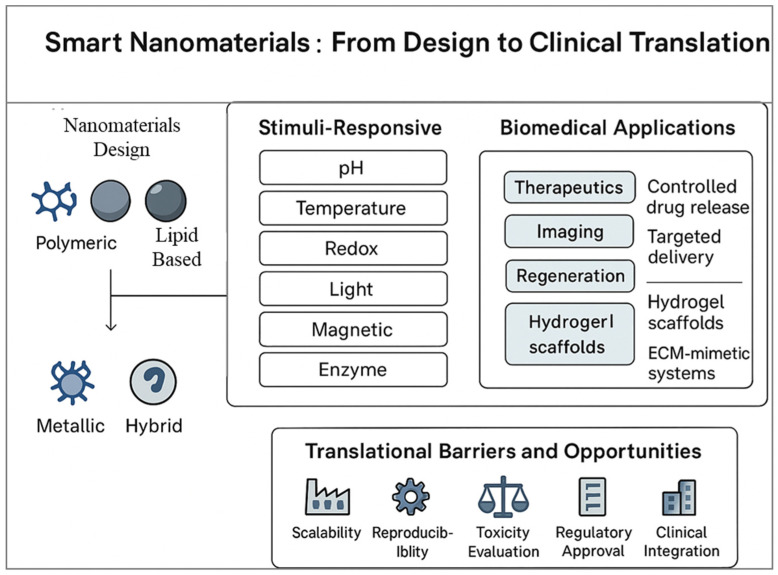
Conceptual roadmap illustrating the design, functionality, and translational trajectory of smart nanomaterials.

Figure 7 presents a conceptual overview of how smart nanomaterials progress from design to clinical translation. The schematic links core nanomaterial classes, polymeric, lipid-based, metallic, and hybrid systems, with their corresponding stimuli-responsive mechanisms such as pH, temperature, redox, light, magnetic, and enzymatic triggers. These mechanisms enable specific biomedical applications across therapeutics, imaging, and tissue regeneration. For example, controlled drug release, targeted delivery, and bioactive hydrogel scaffolds represent key translational outcomes of these materials. The lower section highlights major barriers that currently limit clinical advancement, including scalability, reproducibility, toxicity assessment, regulatory approval, and integration into clinical practice. Understanding these interconnected aspects provides a comprehensive framework for guiding future research toward safer, reproducible, and clinically viable nanomedicine platforms.

## 8. Conclusions

### 8.1. Summary of Key Insights

The development of smart nanomaterials has profoundly influenced modern pharmaceutics by introducing adaptive, precise, and multifunctional platforms for drug delivery, diagnostics, and tissue regeneration. These nanoscale systems exhibit tunable physicochemical characteristics such as controllable size, surface chemistry, and stimuli-responsiveness that enable selective and efficient interactions within biological environments. Through both passive and active targeting strategies, including receptor-mediated uptake and antibody conjugation, smart nanocarriers have achieved superior localization at disease sites while minimizing systemic toxicity. In diagnostics, nanomaterials have advanced molecular imaging and biosensing by enhancing signal sensitivity and allowing real-time monitoring of disease biomarkers. Theranostic nanoplatforms that integrate diagnostic and therapeutic functions exemplify the shift toward personalized and precision medicine. In regenerative medicine, nanocomposite scaffolds, injectable hydrogels, and bioactive interfaces have enabled controlled cell adhesion, proliferation, and lineage-specific differentiation, supporting effective tissue repair. The synergy between material engineering, biological responsiveness, and therapeutic targeting underscores the transformative role of smart nanomaterials in next-generation healthcare.

### 8.2. Outlook on Smart Nanomaterials in Modern Pharmaceutics

Despite extensive progress, the translation of smart nanomaterials from laboratory research to clinical application remains challenging. Key barriers include large-scale and reproducible synthesis, long-term toxicity evaluation, and incomplete understanding of in vivo biodegradation and immune interactions. Standardized regulatory frameworks specific to nanomedicine are still evolving, creating uncertainty in approval pathways. Addressing these issues requires close collaboration between material scientists, biomedical engineers, clinicians, and regulatory bodies to establish validated testing standards and scalable production methods. Future research should prioritize integrating artificial intelligence (AI) and machine learning to guide predictive design and optimization of nanomaterials. These tools can enable real-time modeling of drug release behavior, biodistribution, and therapeutic efficacy, improving precision and reducing development time. Similarly, the combination of smart nanomaterials with 3D bioprinting presents new opportunities for precision tissue engineering, where spatially patterned nanocomposite scaffolds can mimic native tissue architecture and provide controlled biochemical cues. In addition, the incorporation of biosensors and real-time feedback systems can further enhance therapeutic adaptability and safety. Sustainability and biocompatibility will continue to guide material synthesis, emphasizing biodegradable and environmentally safe nanomaterials without compromising efficacy. By uniting smart material design with digital technologies and regenerative approaches, future nanomedicine will evolve toward fully integrated, patient-centered systems capable of dynamic response and long-term therapeutic precision. These advances mark a critical step toward bridging the gap between innovative research and real-world clinical application, paving the way for a new era of personalized, predictive, and regenerative healthcare.

## Figures and Tables

**Table 1:** Comparative overview of stimuli-responsive nanomaterials, their mechanisms, biomedical applications, and limitations.

Stimuli Type	Design/Mechanism	Applications	Key Limitations/Challenges	References
**pH Responsive**	Acid labile bonds (hydrazone, acetal, imine, cis aconityl) cleave in acidic conditions; charge switch polymers gain positive charge in low pH	Tumor targeted drug release, improved intracellular uptake, theranostics using gold or iron oxide nanoparticles	Small pH differences between tissues; risk of premature release in systemic acidosis	[23,24,25,26,27,28,29]
**Temperature Responsive**	LCST type polymers (e.g., PNIPAM) collapse above 32 °C; hydrogels form gel depots at body temperature; thermosensitive liposomes destabilize under mild heat (40–42 °C)	Controlled drug release, injectable depots, cancer therapy (ThermoDox^®^ trials)	Achieving uniform heating in deep tissues; risk of off-target hyperthermia	[16,30,31,32]
**Redox Responsive**	Disulfide or thioketal linkages cleave in high intracellular GSH or ROS environments	Targeted release in tumor cytoplasm; gene and anti-inflammatory therapies	Variations in GSH/ROS levels among patients; potential premature degradation	[34,35,36,37,38,39]
**Light Responsive**	Gold nanostructures convert NIR light to heat (photothermal); light cleavable bonds enable photolysis and gel–sol transitions	On demand release, photothermal therapy, implantable light triggered systems	Limited tissue penetration; possible phototoxicity in prolonged exposure	[40,41]
**Magnetic Responsive**	Iron oxide nanoparticles (IONPs) generate local heat under alternating magnetic fields and act as MRI contrast agents	Theranostic use in magnetic hyperthermia, guided drug delivery, MRI imaging	Requires precise tuning of magnetic field; variable heating efficiency	[16,42,43]
**Enzyme Responsive**	Peptide or polymer linkages cleaved by enzymes such as MMPs, cathepsins, or esterases overexpressed in diseased tissues	Targeted release in tumor and inflammatory microenvironments; tissue specific therapy	Enzyme expression varies across diseases; risk of off-target activation	[45,46,47]

**Table 2:** Comparative summary of biodegradation and clearance pathways of major nanomaterial classes.

Nanomaterial Class	Primary Composition/Examples	Main Biodegradation Mechanism	Primary Clearance Route	Key Translational Considerations	References
**Polymeric Nanomaterials**	PLGA, PLA, PCL, PEG based copolymers	Hydrolysis of ester bonds into lactic and glycolic acids; enzymatic degradation by esterases	Renal or hepatic clearance of soluble degradation products	Widely used in approved drug delivery systems; degradation rate tunable by copolymer ratio and molecular weight	[53,54,55,56]
**Lipid-Based Nanocarriers**	Liposomes, solid lipid nanoparticles, nanoemulsions	Enzymatic hydrolysis by lipases and phospholipases into fatty acids and glycerol	Metabolized via hepatic lipid pathways; minimal long-term accumulation	Excellent biocompatibility and clearance; stability and storage conditions remain key challenges	[57,58]
**Metallic Nanoparticles**	Gold, iron oxide, silver, zinc oxide	Oxidation, dissolution, or surface ligand exchange; partial biodegradation depending on particle size and coating	Phagocytic uptake and sequestration in liver, spleen, and lymph nodes; limited renal clearance for larger (>10 nm) particles	Long-term retention and potential chronic toxicity; requires surface modification for improved excretion	[56,59]
**Hybrid and Composite Nanomaterials**	Metal–polymer or silica–lipid composites	Combined hydrolytic, oxidative, and enzymatic degradation depending on components	Mixed clearance through hepatobiliary and renal pathways	Complexity offers multifunctionality but complicates predictability of degradation and regulatory approval	[60,61]

**Table 3:** Comparative overview of smart nanomaterials for targeted drug delivery: strategies, examples, advantages, limitations.

Targeting/Release Strategy	Nanocarrier Type	Examples/Applications	Key Advantages	Limitations/Challenges	References
**Active Targeting (Ligand–Receptor Interactions)**	Liposomes, polymeric micelles, polymer drug conjugates	Folic acid functionalized nanoparticles for tumors; transferrin modified nanocarriers for proliferating cancer cells; RGD peptide systems for angiogenesis imaging/therapy	High selectivity, improved intracellular uptake, reduced systemic toxicity, adaptable across multiple receptors	Receptor heterogeneity across patients/tumors; ligand density optimization; risk of immune recognition	[11,72,75]
**Active Targeting (Aptamer Functionalization)**	Polymeric nanoparticles, liposomes, gold nanostructures	Aptamer conjugated nanocarriers for PSMA targeted prostate cancer therapy	High binding specificity, non-immunogenic, tunable for different targets	Limited stability in vivo, potential degradation by nucleases	[4,74]
**Antibody Conjugated Nanocarriers**	Liposomes, polymeric micelles, dendrimers, nanoparticles	Trastuzumab conjugated liposomes for HER2+ breast cancer; antibody–nanocarriers for ICAM 1 targeting in inflammation; brain targeting antibodies for CNS disorders	Dual functionality (therapeutic + targeting); strong specificity to well characterized biomarkers	Immunogenicity, high cost, large size reduces deep tissue penetration; scale-up difficulties	[76,77,78,79]
**Passive Targeting (EPR Effect)**	Liposomes, polymeric micelles, albumin bound nanoparticles	Doxil^®^ (liposomal doxorubicin), Abraxane^®^ (albumin bound paclitaxel)	Exploits tumor vasculature leakiness, improved accumulation in tumor tissue, FDA-approved nanomedicines	Heterogeneity of EPR across patients/tumors, limited predictability, poor penetration in dense tumors	[80,83,86,87]
**Stimuli-Responsive Release (Internal Stimuli)**	pH sensitive liposomes, redox sensitive polymeric carriers, enzyme responsive nanoparticles	pH sensitive liposomes releasing drugs in acidic tumor microenvironment; disulfide bond carriers for GSH triggered release; MMP responsive carriers for cancer therapy	On demand drug release at pathological site, higher therapeutic index, minimized systemic toxicity	Complex synthesis, variable pathological conditions across patients, premature degradation risk	[18,88,92]
**Stimuli-Responsive Release (External Stimuli)**	Gold nanorods, SPIONs, carbon nanomaterials, ultrasound responsive carriers	Photothermal therapy with gold nanorods (NIR triggered release); SPIONs for magnetic field triggered targeting; ultrasound triggered carriers	Spatiotemporal control, clinician directed release, useful for precision oncology	Limited tissue penetration of external triggers (e.g., NIR, ultrasound); scalability issues	[21,94,95,96]
**Hybrid Stimuli-Responsive Platforms**	Dual pH–redox responsive polymeric micelles, multi responsive nanoplatforms	Sequential drug release: pH triggered extracellular release + redox triggered intracellular release	Enhanced efficiency, multi-level control, ensures delivery to intracellular targets	Complex synthesis, clinical translation barriers, reproducibility concerns	[97]
**Case Study—Antibody Targeted Liposomes**	Liposomal doxorubicin (trastuzumab modified)	HER2+ breast cancer therapy	Improved tumor selectivity, reduced cardiotoxicity, enhanced therapeutic index	High production costs, immune clearance, variable patient response	[99,100]
**Case Study—Ligand Functionalized Micelles**	Polymeric micelles (folic acid modified)	Paclitaxel delivery in folate receptor–positive cancers	High tumor accumulation, low toxicity to healthy tissues	Dependence on receptor expression, limited scalability	[101,102]
**Case Study—Brain Targeting Nanoparticles**	Transferrin modified polymeric nanoparticles	Alzheimer’s therapy, glioblastoma treatment	Crosses BBB, improves CNS drug delivery	BBB heterogeneity, limited translation in humans	[91,103]
**Theranostic Nanoplatforms (Imaging + Therapy)**	Iron oxide nanoparticles, gold nanorods, polymer drug conjugates	Iron oxide nanoparticles for MRI + drug delivery; gold nanorods for photothermal + chemotherapy	Real time monitoring, multimodal therapy, personalized medicine	High cost, regulatory challenges, potential long-term toxicity	[104,105]
**Case Study—Infectious Disease Targeting**	Mannose functionalized nanoparticles	Anti-tuberculosis therapy (targeting macrophages)	Enhances antibiotic efficacy, reduces systemic toxicity	Variability in pathogen host interactions, immune recognition risk	

**Table 4:** Comparative overview of smart nanomaterials for diagnostic and theranostic applications: strategies, examples, advantages, and limitations.

Strategy/Application	Examples of Nanomaterials	Advantages	Limitations/Challenges	References
**Fluorescence Imaging Nanoprobes**	Quantum dots (QDs), carbon dots (CDs), upconversion nanoparticles (UCNPs), dye doped silica nanoparticles	High sensitivity, photostability, multiplexed biomarker detection, deep tissue penetration (UCNPs)	Possible toxicity (QDs), limited penetration for visible range probes, stability issues in vivo	[6,94,112]
**Biosensing Platforms**	Gold nanostructures, graphene oxide, magnetic nanoparticles	Signal amplification, detection of low abundance biomarkers (ctDNA, miRNA, proteins), real-time monitoring	Risk of nonspecific binding, reproducibility challenges, potential interference from complex biological fluids	[110,111]
**MRI Nanocontrast Agents**	Superparamagnetic iron oxide nanoparticles (SPIONs), manganese oxide nanoparticles, hybrid core–shell nanostructures	High resolution imaging, targeted delivery via ligands, multimodal imaging integration (MRI + fluorescence/photoacoustic)	Safety concerns for long-term accumulation, potential immunogenicity, complex regulatory approval	[116,119]
**Theranostic Nanoplatforms (Oncology)**	Gold nanostructures, mesoporous silica nanoparticles (MSNs), drug loaded SPIONs	Simultaneous diagnosis and therapy, spatiotemporal drug release, reduced systemic toxicity	Stability in circulation, immune clearance, challenges in large scale reproducibility	[21,121,128]
**Stimuli-Responsive Theranostics**	pH sensitive polymer coated QDs, enzyme activated fluorophores, redox responsive nanoparticles	Controlled drug release at diseased sites, dual function imaging + therapy, real-time monitoring	Risk of premature activation, variability in microenvironment stimuli, safety of degradation products	[123,124]
**Applications Beyond Cancer**	Theranostic liposomes for cardiovascular imaging/therapy, PET nanoplatforms for Alzheimer’s	Disease specific imaging, early detection + therapy integration, versatility across multiple disorders	Limited clinical validation, off-target accumulation, translational hurdles for chronic diseases	[126,127]

**Table 5:** Summary of nanomaterials applied in regenerative medicine, highlighting their strategies, therapeutic applications, associated advantages, and key limitations.

Strategy/Application	Examples of Nanomaterials	Biomedical Advantages	Limitations/Challenges	References
**Injectable Hydrogels and Nanocomposite Scaffolds**	Natural polymers (alginate, chitosan, collagen, gelatin), Synthetic polymers (PEG), Nanofillers (graphene oxide, silica NPs, carbon nanotubes, nanoclays, mesoporous silica, polypyrrole, cerium oxide NPs)	Minimally invasive delivery; conformability to defect sites; mechanical reinforcement; controlled release of growth factors (BMPs, VEGF); electroconductivity for neural/cardiac repair; immunomodulation via macrophage polarization; antioxidant protection	Risk of nanoparticle aggregation; potential immune responses; ensuring long-term biocompatibility and biodegradability	[5,102,143,146]
**Nanoparticles for Stem Cell Therapy**	Gold NPs, Mesoporous silica NPs, Polymeric NPs, Magnetic NPs (MNPs, SPIONs), Hydroxyapatite NPs, Bioactive glass NPs, Graphene, Carbon nanotubes, Cerium oxide NPs	Targeted gene/drug delivery; controlled release; stem cell differentiation (osteogenic, neuronal, cardiac); magnetic guidance of stem cells; MRI tracking; topographical/electrical cues; antioxidant and anti-inflammatory protection	Off-target uptake; cytotoxicity concerns; long-term safety and clearance issues; reproducibility in clinical settings	[6,19,95,147]
**Applications in Musculoskeletal and Neural Regeneration**	Nanohydroxyapatite, Carbon based nanomaterials, Metallic NPs, Nanocellulose, Graphene oxide, Electrospun nanofibers, Conductive polymers, Neurotrophic factor loaded NPs	Bone/cartilage regeneration; osteoinductive signaling; antimicrobial activity; chondrocyte proliferation; ECM deposition; neural guidance; sustained neurotrophic release; enhanced electrical activity for axonal regeneration	Mechanical wear in cartilage scaffolds; chronic inflammation risk; limited spontaneous repair in avascular tissues; challenges in precise neural integration	[102,111,151]
**Biomimetic Nanomaterials for Tissue Engineering**	Self-assembling peptide nanofibers, Nanostructured calcium phosphate, Growth factor loaded nanocarriers, ECM mimicking coatings, RGD modified nanofibers	Mimic ECM structure; guide cell adhesion, proliferation, differentiation; osteointegration of implants; adaptive responses to stimuli (pH, mechanical stress); controlled release of signaling molecules; immune modulation (M2 macrophage polarization); improved graft survival	Need for scalable fabrication; immune compatibility in diverse patient populations; maintaining dynamic responsive	[152]

**Table 6:** Comparative data on the clinical translation of nanomaterials: unresolved challenges, innovative strategies, evolving regulatory paradigms, and instructive case studies.

Aspect	Key Issues/Challenges	Proposed Strategies/Solutions	Examples/Case Studies	References
**Safety, Toxicity, & Immunogenicity**	Uncertain biodistribution and clearance (small < 10 nm → renal elimination; large > 200 nm → liver/spleen accumulation). Long-term organ retention (e.g., AuNPs, carbon nanomaterials). Oxidative stress and ROS generation (e.g., ZnO, TiO_2_). Cationic surface charge causing membrane disruption. Complement activation related pseudoallergy (CARPA) and cytokine storms.	Surface modifications (PEGylation, zwitterionic coatings, biomimetic cloaking). Personalized immunotoxicity profiling. Standardized long-term toxicity studies. Development of biodegradable and stimuli-responsive nanomaterials. Use of organ on chip and in silico predictive toxicology.	Doxil^®^ associated with CARPA hypersensitivity. Persistent gold nanoparticles linked to chronic toxicity.	[11,157,159]
**Manufacturing & Scalability**	Reproducibility issues in nanoparticle size, shape, and surface chemistry during scale-up. High costs of materials (e.g., AuNPs, graphene oxide). Instability of lipid/polymer nanocarriers (aggregation, oxidation, hydrolysis). GMP compliance and batch to batch quality control.	Microfluidic synthesis and high-pressure homogenization for reproducibility. Lyophilization with cryoprotectants for storage stability. GMP compliant facilities with advanced nanoparticle characterization. AI-driven process optimization and continuous manufacturing.	Scale-up difficulties in liposomes, polymeric nanoparticles, metallic nanostructures.	[165,168]
**Regulatory Landscape**	Lack of standardized nanomaterial definitions and global harmonization. Complex classification (drug, biologic, device, or combination). Conventional assays fail to predict in vivo behavior. Long approval timelines due to regulatory uncertainty.	Case by case evaluation of physicochemical properties, biodistribution, long-term safety. Adaptive and risk-based frameworks. Early industry–regulator dialogue. Integration of post marketing surveillance and real-world evidence.	Doxil^®^ approved under drug framework. Nanoparticle coated stents classified as combination products.	[171,173]
**Clinically Approved Nanomedicines (Case Studies)**	Successes show clear benefits in pharmacokinetics, safety, or delivery. Failures highlight unpredictable toxicity or lack of efficacy advantage.	Liposomal encapsulation to reduce toxicity. Albumin bound nanocarriers for solubility. Theranostic nanomaterials (dual therapy & imaging).	Doxil^®^: PEGylated liposomal doxorubicin, reduces cardiotoxicity. Abraxane^®^: albumin bound paclitaxel, solvent free with improved tumor penetration. Feraheme^®^: iron oxide nanoparticles, used in anemia and MRI imaging. mRNA COVID 19 vaccines: lipid nanoparticle carriers. Failures: polymeric NPs of camptothecin discontinued due to safety.	

## Data Availability

No new data were created or analyzed in this study.
